# Social interactions between live and artificial weakly electric fish: Electrocommunication and locomotor behavior of *Mormyrus rume proboscirostris* towards a mobile dummy fish

**DOI:** 10.1371/journal.pone.0184622

**Published:** 2017-09-13

**Authors:** Martin Worm, Frank Kirschbaum, Gerhard von der Emde

**Affiliations:** 1 Department of Neuroethology/Sensory Ecology, Institute of Zoology, University of Bonn, Bonn, Germany; 2 Biology and Ecology of Fishes, Faculty of Life Sciences, Humboldt University of Berlin, Berlin, Germany; Universitat Bielefeld, GERMANY

## Abstract

Mormyrid weakly electric fish produce short, pulse-type electric organ discharges for actively probing their environment and to communicate with conspecifics. Animals emit sequences of pulse-trains that vary in overall frequency and temporal patterning and can lead to time-locked interactions with the discharge activity of other individuals. Both active electrolocation and electrocommunication are additionally accompanied by stereotypical locomotor patterns. However, the concrete roles of electrical and locomotor patterns during social interactions in mormyrids are not well understood. Here we used a mobile fish dummy that was emitting different types of electrical playback sequences to study following behavior and interaction patterns (electrical and locomotor) between individuals of weakly electric fish. We confronted single individuals of *Mormyrus rume proboscirostris* with a mobile dummy fish designed to attract fish from a shelter and recruit them into an open area by emitting electrical playbacks of natural discharge sequences. We found that fish were reliably recruited by the mobile dummy if it emitted electrical signals and followed it largely independently of the presented playback patterns. While following the dummy, fish interacted with it spatially by displaying stereotypical motor patterns, as well as electrically, e.g. through discharge regularizations and by synchronizing their own discharge activity to the playback. However, the overall emission frequencies of the dummy were not adopted by the following fish. Instead, social signals based on different temporal patterns were emitted depending on the type of playback. In particular, double pulses were displayed in response to electrical signaling of the dummy and their expression was positively correlated with an animals' rank in the dominance hierarchy. Based on additional analysis of swimming trajectories and stereotypical locomotor behavior patterns, we conclude that the reception and emission of electrical communication signals play a crucial role in mediating social interactions in mormyrid weakly electric fish.

## Introduction

Communication is an integral component in coordinating interactions between individuals, spanning a wide range of social contexts from agonistic behavior to the formation of groups and collective decision making [1, 2]. Communication systems have developed within all of the main sensory modalities used by animals including active sensory systems, such as sonar in bats [3] and cetaceans [4], and the perception of electrostatic fields in weakly electric fishes [5].

Mormyrid weakly electric fish have evolved a unique electro-sensory capability: by emitting pulse-type electric organ discharges (EOD) they use the same signals both for actively probing their environment, i.e. active electrolocation [6], and for communication with conspecifics [7]. Active electrolocation is based on the perception of these self-generated signals through mormyromast electroreceptor organs [8, 9], which are specialized for detecting object evoked amplitude and waveform modulations of the local EODs and are distributed over large areas of the animals’ skin [10, 11]. Electrocommunication is mediated by a different type of electroreceptor organ, the so called knollenorgans [12], which are time-coders that respond very sensitively to the EODs of other electric fish. The input of knollenorgans to the brain is inhibited centrally by a corollary discharge signal during the production of the self-generated EOD [13], demonstrating that the knollenorgan pathway mediates electrocommunication between individuals [14]. The EOD itself is an all or nothing signal, whose waveform reveals information about the signaler’s identity such as species and gender [15], its reproductive state [16] and relative rank in a social hierarchy [17]. However, EOD waveform remains stable on a short to medium duration time scale. In contrast, the inter-discharge intervals (IDI) are highly variable in duration and their temporal sequence can be related to an animal’s current behavioral state [18].

Social interactions among mormyrids are accompanied by stereotypical motor patterns [19], many of which are reminiscent or even identical to those observed during active electrolocation [20]. Activity dependent EOD production may vary in overall *frequency*, with active animals usually discharging at higher rates compared to resting ones [21–23]. In addition, regularizations of interval distributions [21] have been described in the context of active electrolocation [24] and during social encounters [25]. Apart from general variations in overall discharge rate, distinctive *temporal* IDI-patterns, occurring in specific behavioral situations, have been described in several mormyrid species. These include accelerations during aggressive encounters [26–28], double-pulse patterns during territorial behavior [29], as well as 'rasps', which serve as courtship signals [30]. Furthermore, electrocommunication can also result from *interactive* discharge patterns. In certain situations, mormyrids tend to respond to the signals of a conspecific by discharging at a preferred latency of a few milliseconds [31–33]. This so called 'echo response' has been assigned a function selectively in social contexts [34, 35] and active sensing [36], and although its occurrence is very stereotypical, its functional implications are still unresolved. Prolonged periods of phase-locked discharge activity were shown to lead to sequences of mutual EOD synchronizations that can switch between individuals within a group [37].

Since the emergence of classical ethology as a research discipline, so-called ‘dummies’ have been widely used in behavioral biology to identify the essential components of various releasing mechanisms that can trigger stereotypical behavior patterns [38]. In contrast to using living animals as a stimulus, such an approach guarantees repeatability and allows for a standardized experimental protocol. Analogous to the study of acoustic communication, playbacks of electric signals have e.g. been used to relate EOD properties to male fighting potential [39], mate recognition [40]and to decode the communicative value associated with stereotypical IDI-sequences [21, 41–43].

Reproducing central features of living conspecifics by constructing biomimetic fish dummies has made it possible to investigate personality traits and individual preferences in a variety of fish species [44–51]. On a group level, mobile fish dummies have been used to study cohesion and collective decision making in small shoals of three-spined sticklebacks [52, 53] and zebrafish [54], as well as dynamic interactions in shoals of guppies [55]. Weakly electric fish may be particularly suited for studying social behavior in such an approach, since a central feature of their communication–the emission of electric signals—is easily manipulated by electrical playback experiments [49, 56].

By presenting a mobile dummy fish, which is capable of producing EOD playbacks with naturally occurring IDI-sequences of different temporal patterns and overall frequencies to single individuals of the weakly electric fish *Mormyrus rume proboscirostris*, we combined classical dummy experiments with the active production of communication signals in a standardized experimental setup. In a previous study, we provided evidence that the presence of electrical playback signals is the main determinant for the initiation of following-behavior when compared with visual cues and naturalistic motion patterns [49]. Here, we aimed at finding out whether different IDI-sequences influence the likelihood of individual *M*. *rume* to follow after a mobile dummy fish, and whether such sequences can account for different interaction patterns between the mobile dummy and a live fish, both electrically and with respect to locomotor behavior. We hypothesized that if different IDI-sequences contain varying information, which is registered by the receiving animal, we would also observe varying reactions of the fish to the mobile dummy during electric signaling and by corresponding motion patterns. We found that the animals' following-reactions increased when the dummy emitted electrical playbacks, but this was largely independent of the particular playback pattern which was presented. While certain stereotypical signaling responses occurred in all cases, some electrical response patterns of the animals’ varied when the dummy produced different IDI-sequences. For example, the amount of double-pulse displays depended on the playback pattern, although no adoption of the dummies overall IDI-distribution was observed in general. In addition, *M*. *rume* followed the dummy fish in a differing spatial relationship when a playback was presented compared to an electrically silent control, and certain motor patterns were almost exclusively displayed in response to electric signal presentations. These findings support the idea that electric IDI-patterns convey information and can play a role in spatial interactions and cohesion of individuals within groups of weakly electric fish [57–59].

## Materials and methods

### Animals

Eight individuals of *Mormyrus rume proboscirostris* (abbreviated to *M*. *rume* throughout the paper) with standard lengths between 98 mm and 170 mm were used in the experiments. Animals were bred in captivity at the Humboldt University of Berlin and were approximately six years of age at the time of experimentation. Five individuals were unequivocally identified as males by anal fin morphology [60], the remaining three were presumably female. None of the animals had previously been in a reproductive state. All fish were kept in pairs in tanks under tropical conditions (water temperature ~25°C, water conductivity 100–150 μS cm^-1^, light/dark periods 12/12 h), where they were physically isolated by a water permeable barrier, which prevented physical contact but allowed electrocommunication between the individuals. Food was provided in the form of defrosted *chironomid* larvae at least five times a week. All experiments were approved by the Ministry for Environment, Agriculture, Conservation and Consumer Protection of the State North Rhine-Westphalia (MULNV) and were carried out in accordance with the guidelines of German law, with the animal welfare regulations of the University of Bonn, and with the “Guidelines for the treatment of animals in behavioural research and teaching” [61].

### Experimental setup and electrical playback generation

**Fig 1:**
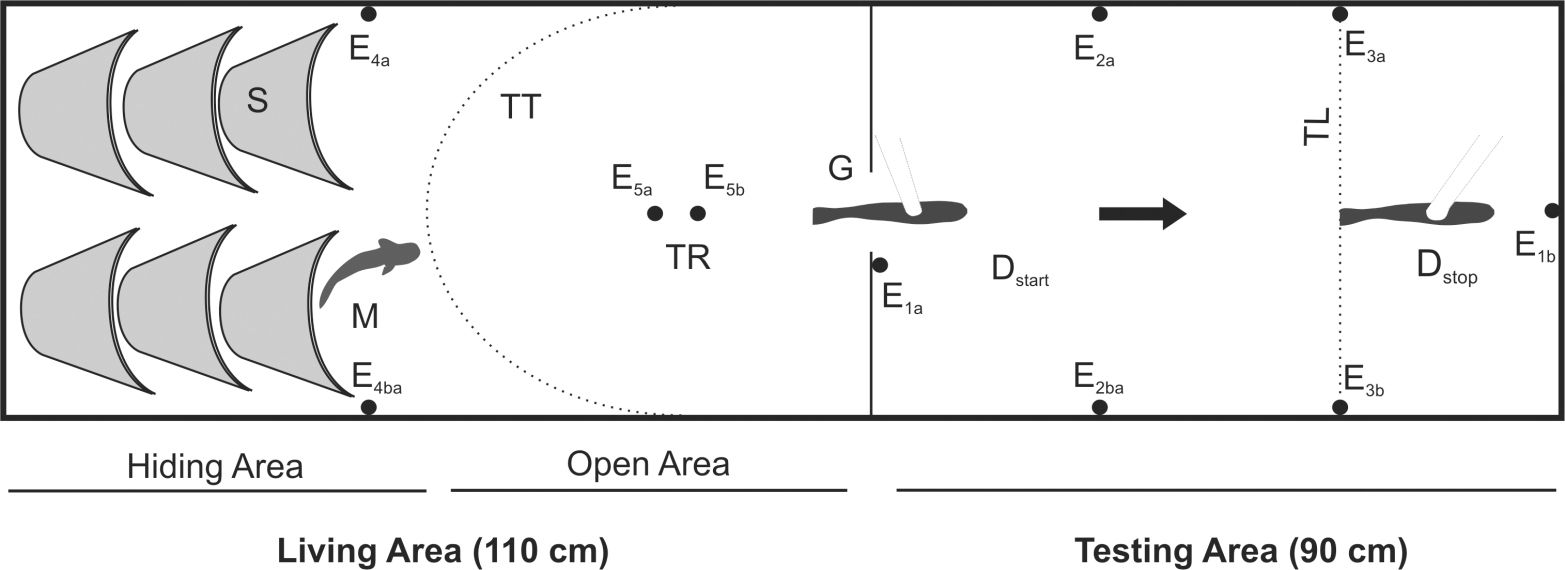
Top view of the experimental tank. S) shelter, M) focal fish, TR) trigger electrodes, TT) approximation of the spatial trigger threshold, G) gate, D_start_) dummy fish at start position, D_stop_) dummy fish at end position, TL) target line defining the following-criterion, E_xa_-E_xb_) Electrode pairs. Figure not drawn to scale. Base area: 200 cm x 50 cm.

Animals were individually transferred to an experimental tank with a ground area of 200 cm x 50 cm and a water level of 20 cm at least one day prior to testing. Water temperature and conductivity were kept constant at 25.2 ± 1.2°C and 100 ± 3 μS cm^-1^ during all experiments. The experimental tank (Fig 1) was subdivided into a 90 cm long testing area and a 110 cm living area, which were connected through a gate that was 10 cm in width. The living area was subdivided into a sheltered area with hiding places in the rear and an open area in front of the gate.

Playbacks consisted of IDI-sequences that had previously been recorded from freely behaving *M*. *rume* and were concatenated from a pre-recorded EOD waveform of a live specimen presented at a sampling rate of 48 kHz. A total of seven playback sequences were used (see S1 Table for detailed descriptions). Playbacks were characterized as either being based on patterns (P) or frequencies (F), with numbers indicating increasing average IDI-duration. They were recorded from fish that were foraging (F_1_), hiding (F_4_) or displaying aggressive behavior in a group (P_A_) [23], following an electrically silent dummy fish (F_2_), slowly swimming (F_3_) or in a subordinate position displaying periods of electrical silence (P_S_), as well as a double-pulse pattern containing alternations of long and short IDIs (P_D_).

A dummy fish was made from a 120 mm black fishing bite (Kopyto-Relax) that was endowed with a pair of carbon electrodes separated by a distance of 90 mm along its longitudinal axis. The dummy fish was attached to a white plastic rod that was connected to a slide, which could be moved along a track above the testing area of the experimental tank. In order to establish standardized experimental conditions with a similar relationship of the fish's initial behavior and the activity of the mobile dummy, the onset of every experimental trial was triggered by an EOD of the tested fish. This was accomplished by burying a pair of trigger electrodes within the open area of the living compartment (TR in Fig 1). Differential amplification (Brownlee Precision Model 440, Palo Alto, CA) of the signal measured between these electrodes defined an area between the hiding area and the gate (TT in Fig 1), where an EOD exceeding a predefined threshold value initiated the start of an experimental trial (see below) via a TTL-pulse (Transistor-Transistor Logic), which was generated by a digital oscilloscope (Yokogawa DL1620, Yokogawa Electric Corp., Tokyo, Japan). The threshold was adjusted for each fish by adjusting the amplification according to the magnitude of the fish's EOD. This configuration started an experimental trial when the fish produced an EOD after leaving the hiding area. The TTL pulse initiated the execution of an experimental sequence via Spike2 (Version 5.21, Cambridge Electronic Design, Cambridge, UK) starting both movement and electric signal generation of the dummy fish. The respective playback sequences were sent to the dummy via a D/A-converter (CED Power 1401, Cambridge Electronic Design, Cambridge, UK) and an analog stimulus isolator (model 2200, A-M Systems Inc., Carlsborg, WA) capable of reproducing the natural EOD-waveform of *M*. *rume*. The resulting electric field (see [49] for a characterization in a similar dummy), measured head-to-tail very close to the dummy, had an amplitude of 19.05 V_p-p_ and thus was slightly stronger than that produced by the largest test fish (13.87 V_p-p_). A DC motor was used to move the slide with the dummy via a cord linkage, thus moving the dummy fish through the testing area at a speed of 0.11 m s^-1^. Two control conditions were performed *without* electrical playback. In one, only the moving dummy fish was presented (C_1_), while in the other (C_2_) the dummy remained motionless at the end position D_stop_ (compare Fig 1).

All experiments were performed in complete darkness with only infrared light illumination (850 nm, IR Illuminator Model SA1-60-C-IR,Itakka, Wattens, Austria), which is invisible for the fish [62]. Both the living area and the testing area were monitored with infrared-sensitive video cameras (DBK 21AF04 FireWire Camera with Vari Focal T4Z2813CS-IR CCTV Lens, The Imaging Source, Bremen, Germany) from above.

### Experimental protocol

The trigger mechanism for starting an experimental trial was activated only when the test fish sojourned in the hiding area. Once an experimental sequence was initiated by the test fish, the dummy fish moved across the testing area for 7.5 s while either emitting one of the seven playback patterns or remaining silent during control (C_1_). No movement was induced during control condition C_2_.

For each animal, two experimental sessions were conducted on non-consecutive days, during which a total of ten successful presentations of each playback sequence were given in random order to each animal. For all but the second control condition (C_2_), a presentation was defined to be a successful following-trial, if the test fish followed the dummy across an imaginary target line perpendicular to the rear end of the dummy fish at its end position (TL in Fig 1) within 15 seconds after the trial was initiated. Playback conditions were presented in randomized order with inter-trial intervals of at least 10 minutes. Non-successful presentations were repeated. In order to get the test fish accustomed to the treatment and avoid the possibility of ceiling effects [63], each experimental session was preceded by a series of ten trials during which a regular 20 Hz playback sequence was used as a stimulus, which was within the range of natural discharge frequencies displayed by *M*. *rume*, but different from all the playback patterns used during the actual experimental trials. Relative following-scores were calculated for all eight fish by dividing the number of presentations during which the following-criterion was met by the total number of trials of the respective experimental condition.

### Data acquisition

Electric signals were recorded via an array of five pairs of silver electrodes mounted in the experimental tank, which were arranged orthogonally in order to account for all EODs independently of the fish’s position in the tank. All signals were amplified, digitized and recorded in Spike2 for subsequent analysis as time series. Simultaneously, all activity in the testing area was recorded to disk at 15 fps. Data were recorded during 30 s following the trigger signal.

### Hierarchy determination

In order to determine the relative hierarchy of all individuals, animals were transferred pairwise into an illuminated tank with a white ground area of 60 cm x 30 cm. The single shelter provided was a 20 cm x 5 cm transparent red plastic tube. The animal that acquired ownership over the tube after 20 minutes was considered to rank higher than its opponent. Ownership was expressed either by occupying the tube or by aggressively preventing the opponent from doing so (compare [64]). Each fish was tested against all other fish in successive contests. In order to mitigate potential effects of the outcome of previous contests on the following encounter [65], no individual was tested more than once per day. Standard length and body weight of all animals were subsequently determined by placing each individual on laminated scale paper and weighing them wrapped in moist tissue.

### Locomotor behavior

A total of seven different motor behavior patterns were quantified from the video recordings, which were randomized to rule out observer bias during the analysis. A 'cut off' occurred when the test fish intercepted the dummies' swimming trajectory and crossed its pathway during the first 7.5 seconds after onset of the experiment. 'Circling' [26] was defined as a full circle by the test fish around the dummy during the first 15 seconds of an experiment. Incomplete circles within the same time frame were counted as 'lateral probing' [19, 20]. 'Lateral va-et-vient' comprised short forward and backward swimming movements at a constant distance to the dummy and 'radial va-et-vient' consisted of small tail strokes directed towards the dummy after a turn of 180° [20]. 'Lateral va-et-vient' was only quantified between seconds 7.5 and 15, when the dummy had already stopped moving. A 'head butt' occurred when the test fish hit the dummy by a strike with its head [28, 31], while instances of 'touch' lead to visible deflection of the dummy fish by physical contact without obvious aggressive intent.

### EOD data analysis

Recorded EOD data were reduced to time series, and the signals of the fish and the playback were separated for further analysis (S1 Data). Data from the 10 replicated trials per experimental condition of the same individual were pooled for histogram representation and averaged for subsequent statistical analysis of distribution parameters to avoid pseudo-replication due to repeated experimental conditions with the same individuals. The autocorrelation of a fish's discharge sequence was used to quantify the amount of regularization. Adaptive cross-correlations between playback signals and EOD responses were calculated to quantify electric discharge synchronizations of *M*. *rume* with the mobile dummy fish. These analyses were performed according to the procedure described in [23]. In short, IDI-sequences of fish and playback were transformed to high-resolution time series using exponential filtering. Pearson’s correlation coefficients were then determined over the experimental time for a ‘response time’ of 100 ms between the two time series. The maximum cross-correlation value within this 100 ms time window was then extracted for the electrical reaction of *M*. *rume* to the playback sequence from seconds one to 14. Data were averaged over a duration of 1/15 seconds to obtain a single value per video frame. The relative amount of correlation between the fish's signals and the playback signals was then compared for the different playback conditions. In addition, the duration of sequences of video frames with correlation coefficients greater than 0.3 was quantified. The amount of random cross-correlations between playbacks and fish responses was assessed by running the analysis using IDI-sequences emitted by the fish during the moving control condition C_1_ for each playback. A generalized linear mixed model (GLMM) using repeated measures of each playback and individual fish as fixed factors was used to assess the overall statistical difference between random correlations and those resulting from discharge interactions with electrical playback patterns.

Double-pulse patterns were defined as sequences of alternating long and short IDIs. The minimum definition used for the quantification of a double-pulse pattern in this study was a sequence of at least five consecutive IDIs, where intervals 1, 3 and 5 were ≥ 60 ms and intervals 2 and 4 were ≤ 50 ms. Analysis was performed automatically using a custom written Matlab script (Version R2013b, The MathWorks Inc. Natick, MA).

Echo-responses were analyzed by quantifying the relative occurrence of latencies with which each playback EOD was followed by EODs of the fish. These latencies were compared to the distribution that would be expected if the IDI-sequences of playback and fish were two independent time series. Echo responses were quantified according to [31] by calculating the ratio of observed to expected latencies at the mode of the observed latency distribution.

Statistical comparisons between experimental conditions were performed in IBM SPSS Statistics for Windows (Version 22.0, IBM Corp., Armonk, NY) using repeated measures ANOVA if data were assumed to be normally distributed as assessed by the Shapiro-Wilk test. In cases where the assumption of sphericity was violated according to Mauchly's test, epsilon (ε) was used to adjust the degrees of freedom according to Greenhouse and Geisser [66]. Data not meeting the criterion of normality were analyzed using the non-parametric Friedman's two-way analysis of variance by ranks. Associations with hierarchy rank were determined based on Spearman rank correlations (*ρ*). Statistical significance was accepted at the *α* = 0.05 level.

### Video tracking

For comparison of swimming-trajectories dependant on the presence or absence of electrical playback signals, all videos recorded for playback condition F_2_ and the control C_1_ were rectified to correct for radial distortion and subsequently tracked to obtain trajectories and spatial orientations for both the dummy and the focal fish. Tracking was performed using Ctrax [67] including the provided Matlab toolboxes for subsequent correction and analysis of tracking data. The distance between test fish and the dummy was determined for each frame as the shortest connection between the snout of the test fish and any point on the ellipse representing the dummies current position. The angular relationship between dummy and fish was determined from the dummy's coordinate system by calculating the absolute angle between the dummy’s orientation and the line connecting the centers of the ellipses representing fish and dummy. The average cross-correlation coefficients between electric signal sequences and the temporal occurrence of double pulses were then assigned to each frame. In order to guarantee synchronicity between EOD- and video recordings, an infrared LED was activated simultaneously with playback presentation and recorded on video.

## Results

### Dominance hierarchy

**Fig 2:**
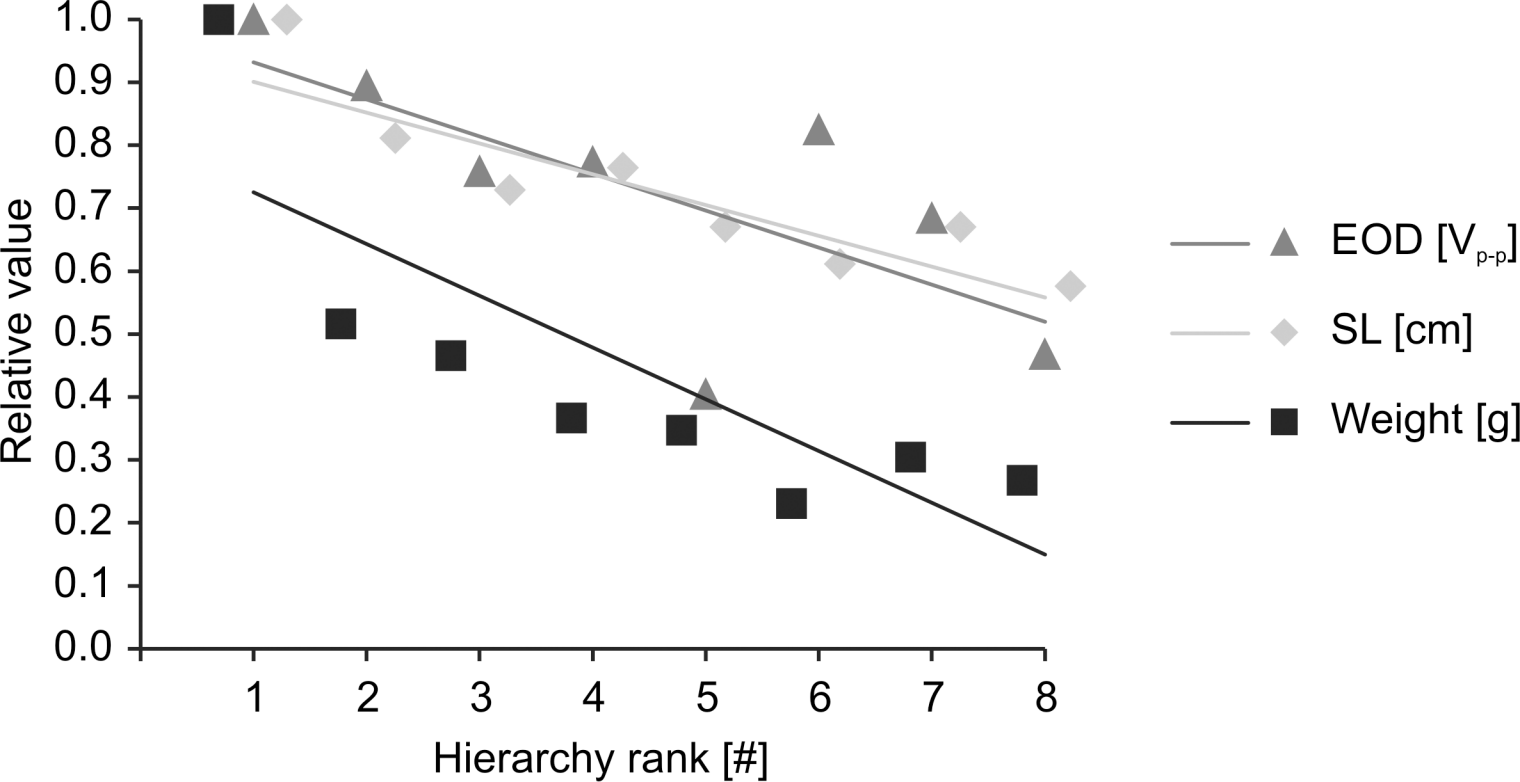
Associations between hierarchy rank and fish characteristics. EOD-amplitude, weight and standard length increased with increasing position within the hierarchy, where #1 is the highest and #8 the lowest ranking individual.

Based on hierarchy experiments, all eight tested animals could be unequivocally assigned to a relative dominance rank within the group, with fish #1 being the highest and fish #8 the lowest ranking individuals. Increase in hierarchy rank was correlated with an increase in the animals' standard length (*ρ*_*s*_ = -0.934, *p* = 0.001), weight (*ρ*_*s*_ = -0.929, *p* = 0.001) and peak-to-peak EOD amplitude (*ρ*_*s*_ = -0.714, *p* = 0.047) (Fig 2).

### Following-behavior

**Fig 3:**
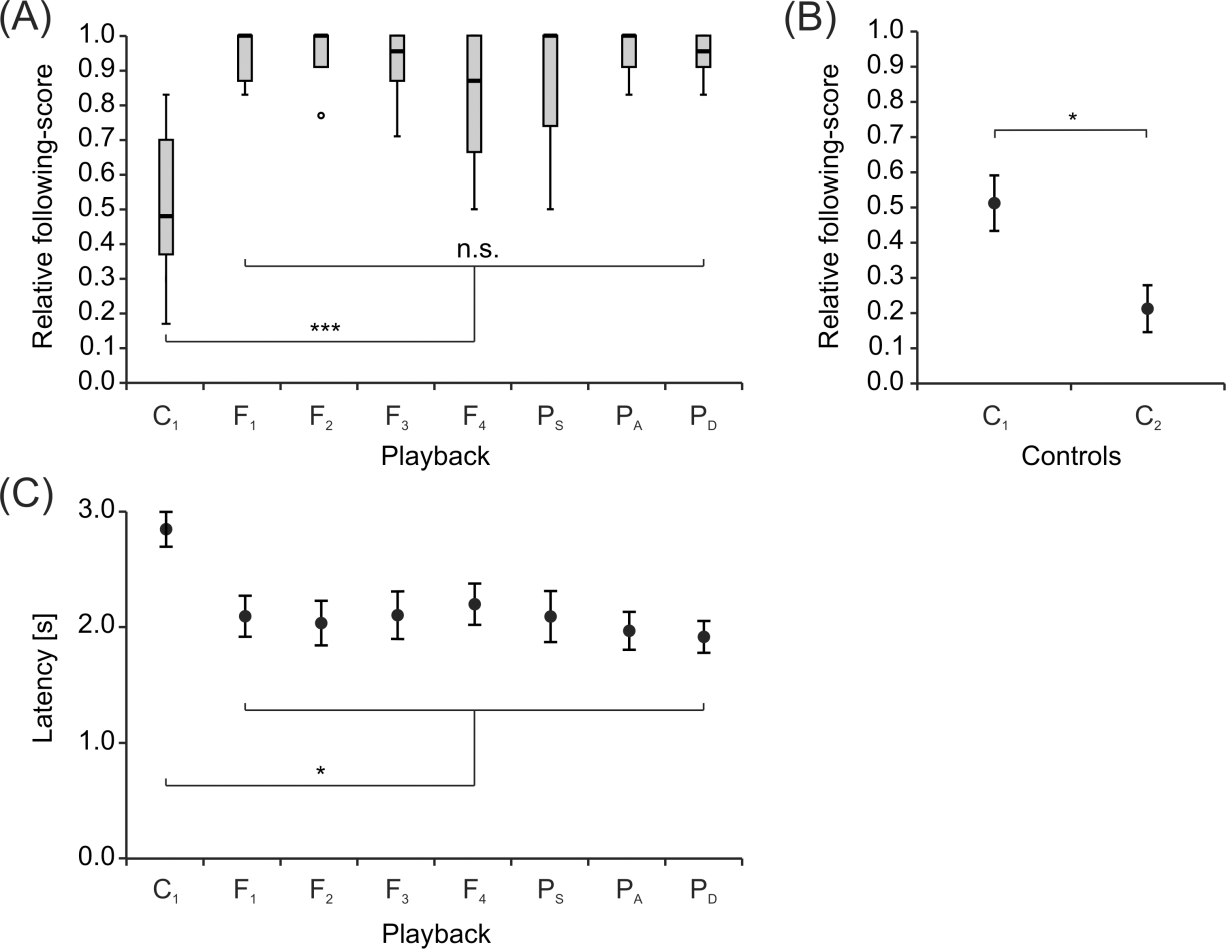
Following-behavior based on playback presentation. Different playbacks are given on the abscissa. (A) Box plots of relative following-scores for single *M*. *rume* following a mobile dummy fish. Animals follow more often during playback presentation compared to the control condition (C_1_). (B) Comparison of relative following-scores (*mean ± s*.*e*.m) between the control conditions. Animals cross the target line more often, if the electrically silent dummy moves across the testing area (C_1_). (C) Latency (*mean ± s*.*e*.m) of fish to enter the testing area after onset of the experiment, i.e. after the dummy fish started moving.

Analysis of relative following-scores (Fig 3A) revealed a statistically significant difference between the treatments (*χ*^2^(7) = 30.517, *p* < 0.001) with all conditions involving electrical playback forming a homogenous subgroup (*χ^2^* = 3.442, *p* = 0.752). Single individuals of *M*. *rume* were therefore less likely to be recruited into the testing area by an electrically silent dummy compared to a dummy emitting EODs (*median* score = 0.48). However, there was no overall effect on following-behavior in response to the different playback sequences (*median* scores: 0.87–1).

In order to test whether animals would enter the testing area and meet the following-criterion independently of the experimental conditions, no stimuli were presented after activation of the trigger during control condition C_2_. Statistical analysis (paired-samples t-test, *t*(7) = 3.267, *p* = 0.014) confirmed a significant difference of relative following-scores between the control conditions C_1_ (*mean ± s*.*e*.*m*. = 0.51 ± 0.08) and C_2_ (*mean ± s*.*e*.*m*. = 0.21 ± 0.07) (Fig 3B), indicating that following-behavior did not occur spontaneously, but instead was triggered by the movement of the dummy fish, even when the dummy was electrically silent.

There was a statistically significant effect of experimental condition on the animals' latency to enter the testing area (*F*(2.912, 20.385) = 11.210, *p* < 0.001, ε = 0.416) (Fig 3C). Without playback, animals took on average 0.79 ± 0.17 (*mean ± s*.*e*.*m*) seconds longer to enter the testing area as indicated by a Bonferroni adjusted comparison (*p* = 0.014) between the control C_1_ (*mean ± s*.*e*.*m*. = 2.85 s ± 0.16 s) and the average of all conditions featuring electrical playback. Latencies for the conditions featuring electrical playback did not differ statistically (*F*(6, 42) = 1.828, *p* = 0.117)).

A positive correlation between hierarchy rank and relative following-scores was observed in all eight individuals (Table 1), which was significant for the control condition C_1_ (*ρ*_*s*_ = 0.976, *p* < 0.001) and the low frequency playback F_4_ (*ρ*_*s*_ = 0.781, *p* < 0.022), meaning that in these situations higher ranking individuals were more likely to follow the dummy than lower ranking fish.

**Table 1:** Associations between dominance rank and following-behavior.

Playback	ρ_s_	*p*-value
C_1_	0.976	**< 0.001**
F_1_	0.124	0.77
F_2_	0.316	0.446
F_3_	0.524	0.183
F_4_	0.781	**0.022**
P_S_	0.436	0.28
P_A_	0.357	0.385
P_D_	0.483	0.226

Spearman rank correlation and corresponding *p*-values between dominance rank and relative following-score according to playback condition.

### Electrical responses

**Fig 4:**
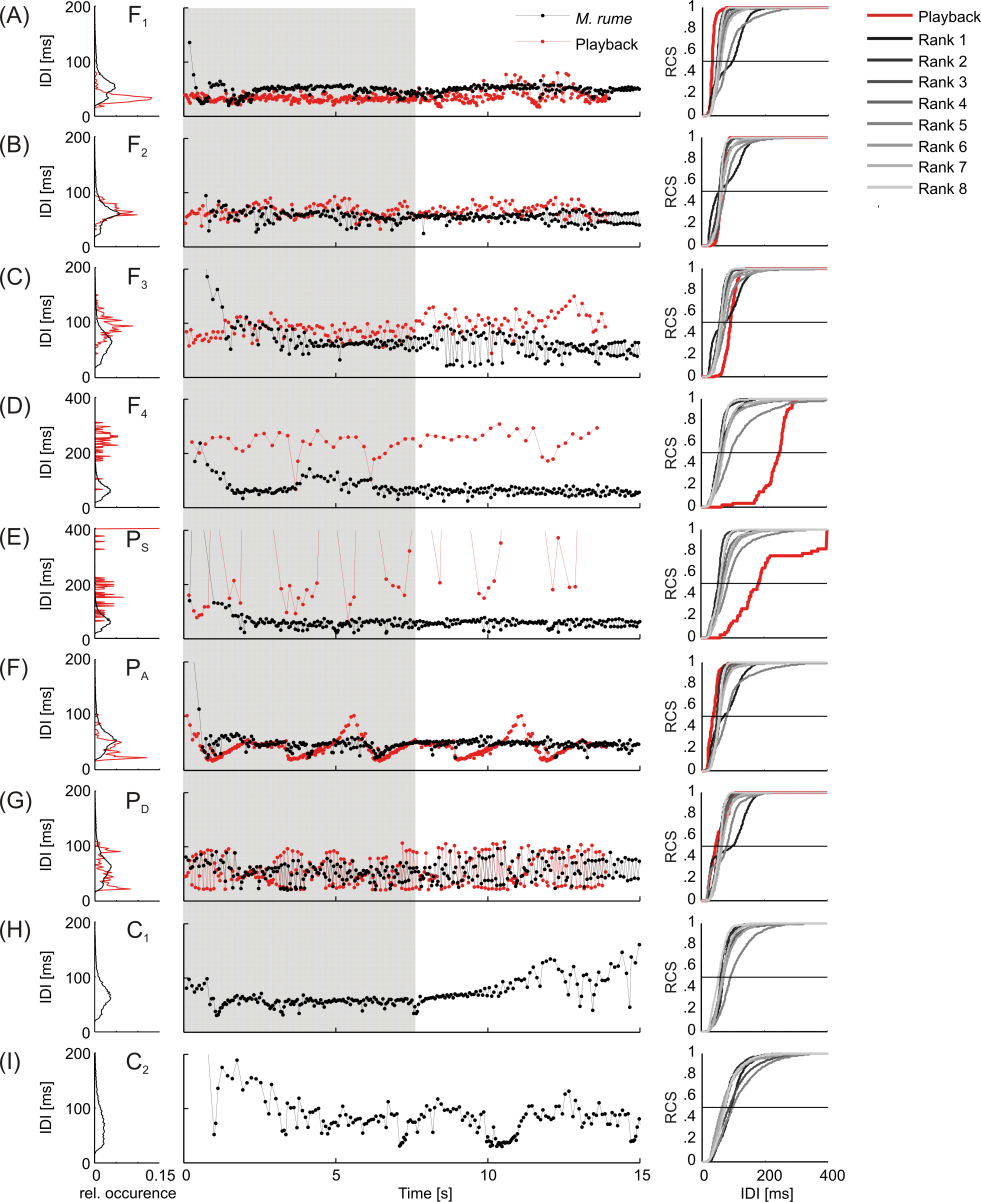
Representation of playback conditions and the electrical responses of *M*. *rume*. Left: Relative IDI-distribution of playbacks (red) and *M*. *rume* (black) pooled for all individuals per condition. Middle: Time course of electrical playback IDIs (red) with an exemplary response of fish #2 (black). Right: Relative cumulative sums (RCS) of IDI-distributions of playbacks (red) and *M*. *rume* (grey, graded to distinguish between different ranks). Each curve represents data from ten trials that were recorded from an individual fish within the respective condition. The shaded area represents the duration of dummy movement. Note different scaling in D and E.

The electrical responses of *M*. *rume* to the different playback and control conditions are summarized in Fig 4. In the central column, IDI-duration is plotted versus experimental time for all playbacks presented (red), and a representative response of fish #2 (black), to demonstrate the patterning of the respective signal sequences. The relative occurrence of interval lengths and their distribution is depicted on the left hand side of Fig 4 for the presented playbacks (red) and the summed electrical responses of all *M*. *rume* to the respective experimental conditions (black). Statistical comparison of IDI-distribution parameters for 15 s sequences averaged over the ten trials performed with each individual fish per experimental condition revealed significant differences between IDI mean (*χ^2^*(8) = 36.167, *p* < 0.001), IDI median (*χ^2^*(8) = 29.467, *p* < 0.001), IDI mode (*χ^2^*(8) = 21.378, *p* = 0.006) and the inter-quartile difference (q75-q25, *χ^2^*(8) = 26.933, *p* = 0.001, see supplementary S1 Fig for details). The same data are plotted for each fish separately as relative cumulative sums (RCS) on the right hand side of Fig 4, which allows assessing the contributions of individual *M*. *rume* to the overall IDI distribution in each category. Evidently, animals did not adopt the overall IDI-distribution featured by the playback emitted by the moving dummy fish. Instead, IDI-distribution modes were approximately the same for the electrical responses to all playbacks, including the silent control C_1_ and were most reminiscent of the IDI-distribution in playback F_2_, with a mode at 64 ms (Fig 4B). An exception is represented by the motionless control condition C_2_ (Fig 4I), where animals discharged less regularly and with longer intervals leading to a broader IDI-distribution. In addition, it becomes evident from the cumulative histograms, that electrical discharge responses were not uniform across individual fish. Particularly for the highest ranking individual fish #1, a second turning point in the histogram indicates a bimodal IDI-distribution in response to all but the low frequency playbacks F_4_ and P_S_ (Fig 4D and 4E) and the controls C_1_ and C_2_ (Fig 4H and 4I).

**Fig 5:**
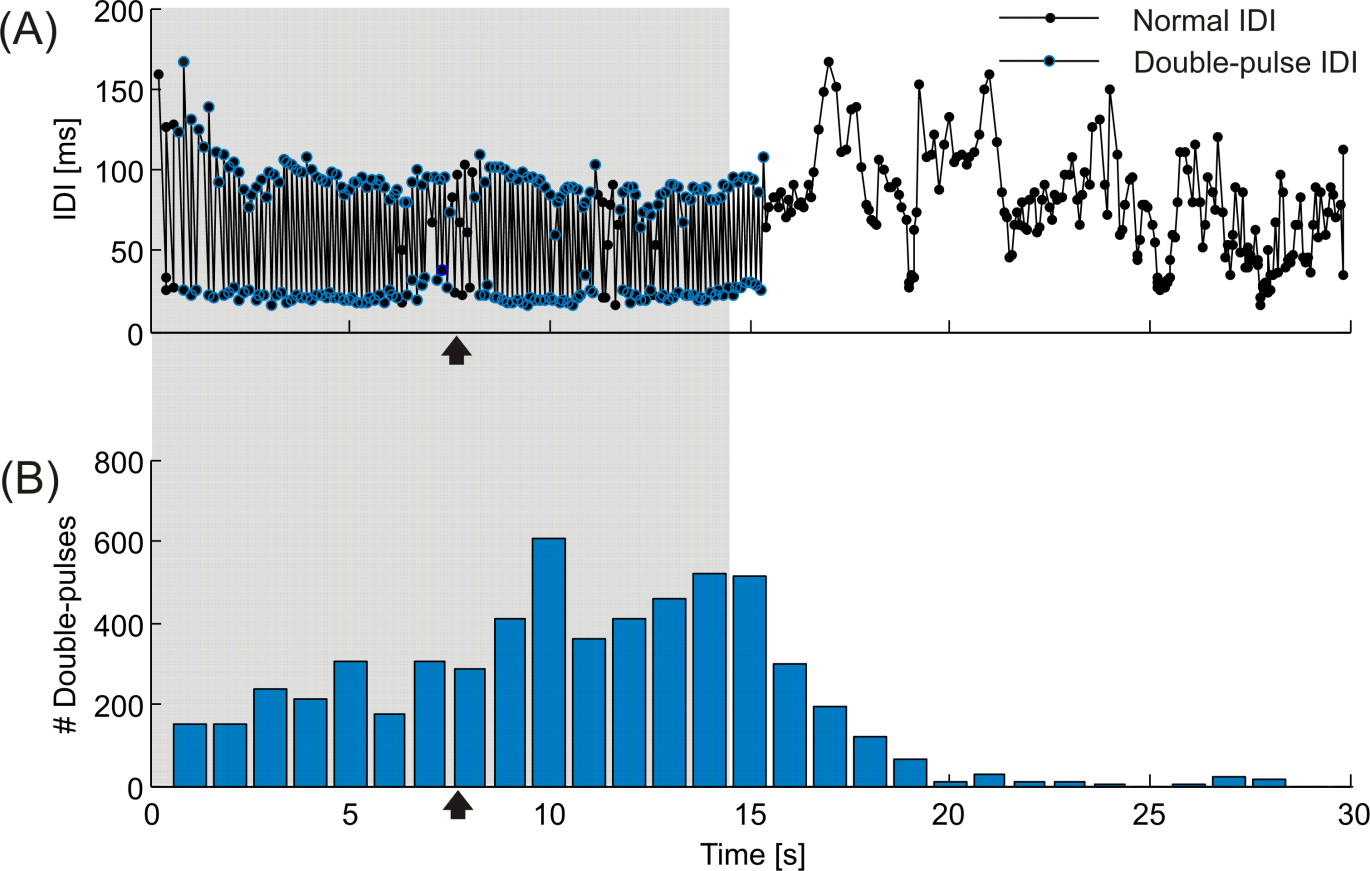
Double-pulse responses over time. (A) Exemplified electrical response of fish #5 to the double-pulse playback P_D_ (see Fig 4G) with intervals belonging to double-pulse sequences marked by blue circles. (B) The total amount of double-pulse related IDIs is pooled per second for the time course of all experimental trials with playback P_D_. The shaded area represents the duration of the playback. Dummy fish movement stopped at the time points indicated by black arrows.

**Fig 6:**
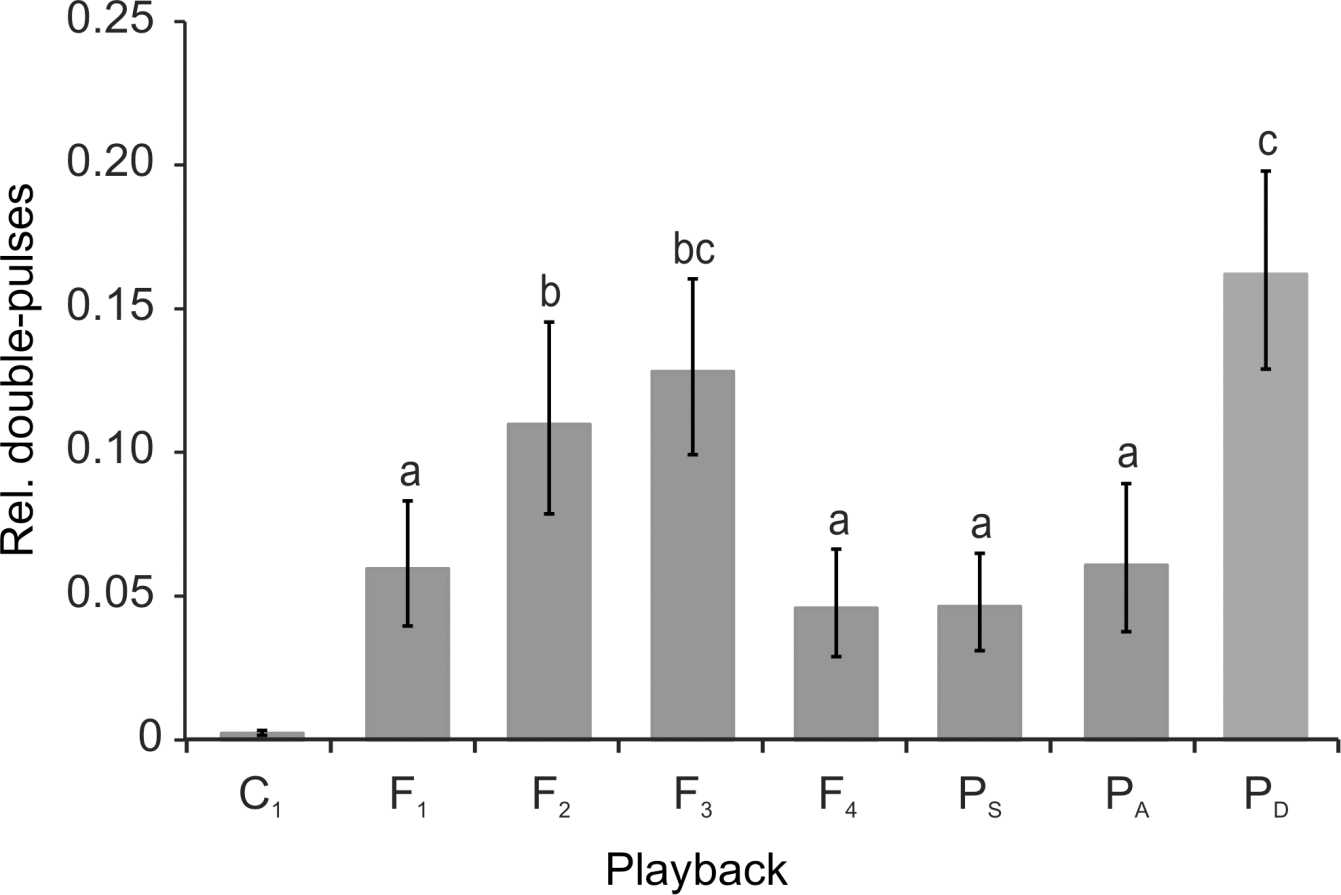
Double pulses in response to electrical playbacks. Relative amount (*mean ± s*.*e*.m) of double pulses emitted in response to different playback conditions and the electrically silent control (C_1_). Categories not sharing a common superscript letter differ significantly based on Fisher’s LSD (*α* = 0.05).

A particular discharge pattern was represented by double pulses, which were sequences of alternating long and short IDIs. Fig 5A shows an exemplary double-pulse pattern displayed by fish #5 in response to playback P_D_, which also featured double pulses (Fig 4G). The temporal occurrence of double pulses in response to all experimental trials featuring playback P_D_ is summed over the recording period of 30 s in Fig 5B, and demonstrates a steep decline of this pattern within a few seconds after the end of playback presentation. The amount of double pulses varied between the different playbacks. They were most numerous in response to the double-pulse playback P_D_, differing significantly from all but the response to playback F_3_ based on Fisher's LSD (*F*(3.070, 21.488) = 18.351, *p* < 0.001, ε = 0.439 on arcsine-square-root transformed data). A functional role of double pulses as a communication signal is supported by the fact, that this pattern was virtually absent during the silent control condition C_1_ (Fig 6).

**Fig 7:**
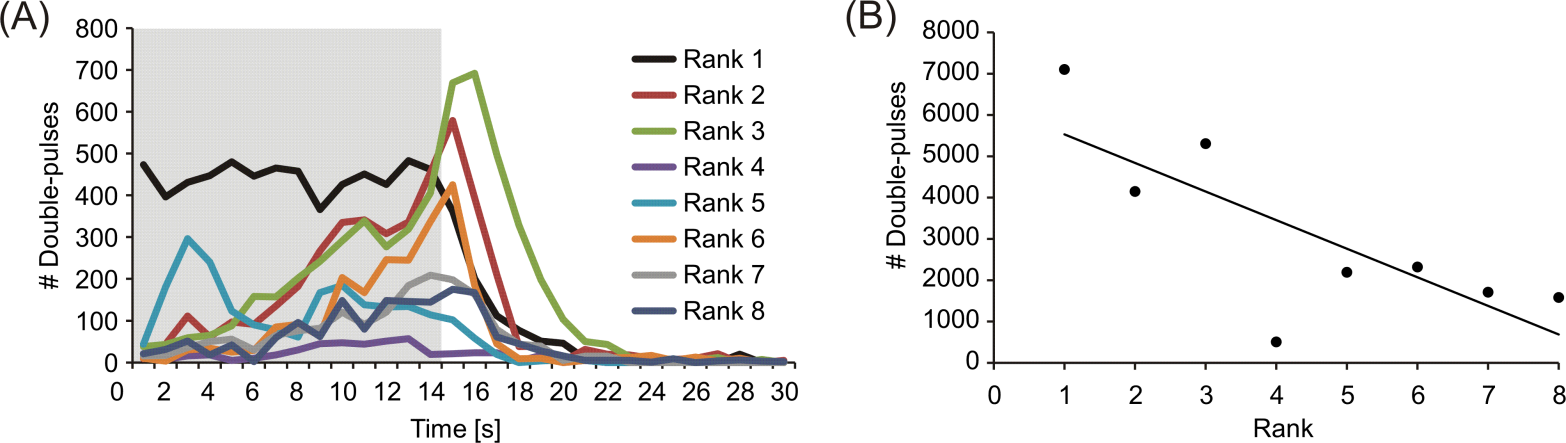
Double pulses and hierarchy rank. (A) The amount of double pulses emitted per second of experimental time by each fish is summed for all trials involving electrical playback presentation. Individual fish are color coded according to their hierarchy rank. The shaded area represents the duration of the playback. (B) Association between double-pulse display and hierarchy rank for all tested individuals of *M*. *rume*.

**Fig 8:**
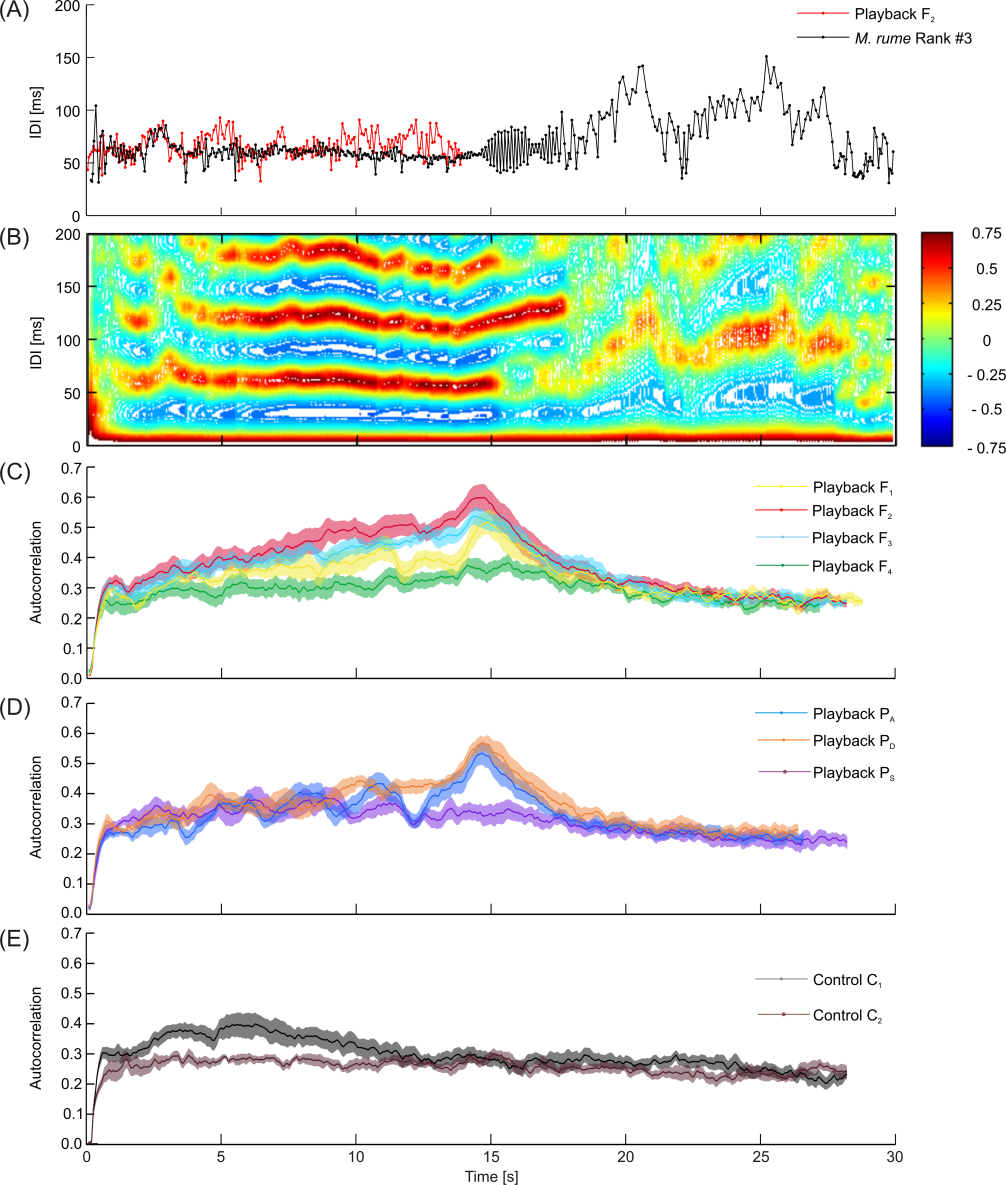
Autocorrelation of electric discharge activity in *M*. *rume*. (A) Exemplary demonstration of a regular discharge pattern with high autocorrelation (compare B) of fish #3 (black) in response to playback F_2_ (red). Note the typical double-pulse pattern short after stimulus offset. IDIs are longer and more variable in the second half of the recording, when the dummy was silent. (B) Autocorrelation diagram for the sequence shown in (A), with a color coded representation of the correlation between the fish's current discharge activity with its own signal within the previous 200 ms. (C-E) Maximum autocorrelation for all trials averaged for all individuals of *M*. *rume* depicted over a time period of 30 seconds. (C) Frequency-based playback trials F_1_-F_4_; (D) pattern-based playbacks P_A,D,S_; (E) controls C_1_ and C_2_. Shaded areas represent the standard error of the mean.

Apart from differences in the amount of double-pulse discharges in response to different electrical playbacks, there was also a variation in double-pulse displays as a general response to the presentation of electrical playback among individual fish. Fig 7A sums the total amount of double pulses over time that was emitted by each individual of *M*. *rume* in response to all trials featuring electrical playback. Similar to the data presented in Fig 5B, double-pulse production increased in most fish over the time course of playback presentation, peaking shortly after its offset (see also Fig 8A) and declined to virtually zero within a few seconds afterwards.

The total amount of double pulses displayed by an individual fish was furthermore correlated with its rank within the hierarchy, with higher ranking individuals producing more double pulses than lower ranking ones (*ρ*_*s*_ = -0.714, *p* = 0.047, Fig 7B). In addition, the number of double pulses produced within an experimental trial generally decreased with the number of trials performed with an individual in an experimental session, indicating that this signal pattern was subject to habituation. In response to playback F_2_, the highest amount of double pulses was emitted at a distance of approximately 100 mm between *M*. *rume* and the dummy fish, and none were observed at a distance greater than 287 mm.

Autocorrelation coefficients of discharge sequences were calculated in order to quantify discharge regularizations, with higher coefficients pointing to more regular discharge activity in *M*. *rume*. The average maximum amount of autocorrelation within a time frame of 200 ms over the recording period of 30 seconds was highest for playback F_1_ (*mean* = 0.352, 95% CI [0.299, 0.405]) and lowest for the stationary control C_2_ (*mean* = 0.265, 95% CI [0.233, 0.296]). No experimental category differed significantly from the moving control C_1_ (*mean* = 0.327, 95% CI [0.272, 0.382]), based on Bonferroni adjusted *p* = values (see S2 Fig for detail). Fig 8 summarizes the quantification of autocorrelation within a signal sequence over time. An exemplary IDI-sequence of fish #3 (black) with strong regularization in response to playback F_2_ (red) is depicted in Fig 8A. The animal responded to the offset of the playback stimulus with a short sequence of double pulses, and continued to discharge with longer and less regular intervals for the rest of the recording. For the sequence depicted in 8A, autocorrelation is quantified over time in Fig 8B, with correlation coefficients color coded from -0.75 to 0.75 for the timeframe analyzed. Autocorrelation within the discharge activity of fish #3 was strong during playback presentation and the short sequence of double pulses that followed, and decreases abruptly thereafter.

Average time courses of regularization of all fish in response to playback and control conditions are depicted in Fig 8C–8E. Data are mean values of the average autocorrelation displayed per frame by all fish in the respective experimental category, with shaded areas representing standard errors of the mean. During electrical playback presentation, correlation coefficients steadily increased, peaking shortly after the offset of the stimulus and then declined to a baseline level of approximately 0.3, similar to the value of the motionless control C_2_. This effect was weaker or even absent in response to the low frequency playbacks F_4_ and P_S_ (Fig 8C and 8D). The moving control C_1_ caused an initial short increase in regularization that declined a few seconds afterwards and reached baseline levels after the dummy fish stopped moving. Quantification of the duration of coherent sequences of autocorrelation exceeding the baseline level of 0.3 revealed longer sequences in response to higher frequency playbacks as compared to the low frequency playbacks F_4_ and P_S_ and the controls (S3 Fig).

### Electrical discharge interactions and synchronizations

**Fig 9:**
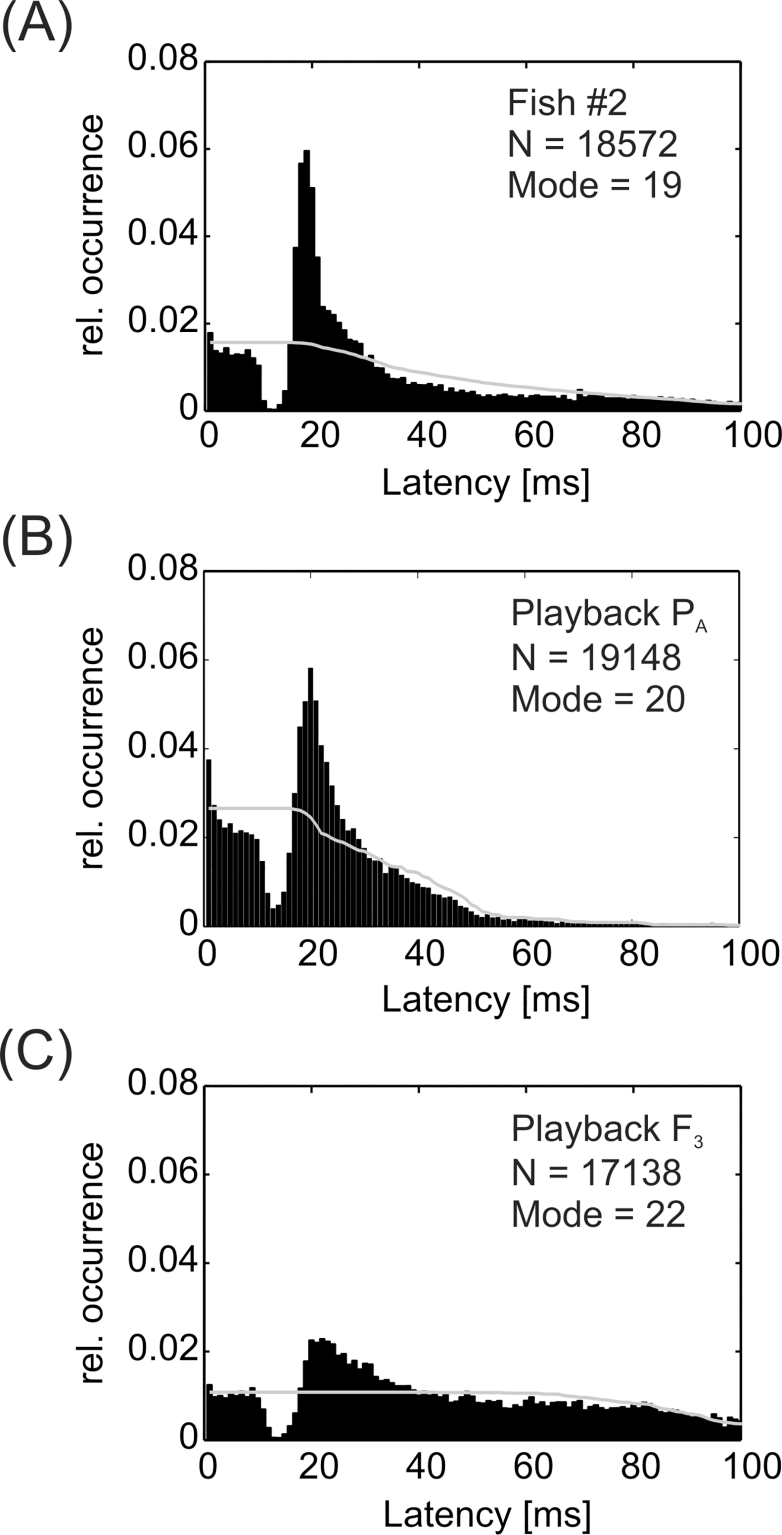
Echo responses in *M*. *rume*. A combination of preferred latencies and latency avoidance occurred in all individuals and in response to all electrical playbacks. Overall results are pooled exemplarily for the responses of male fish #2 (A) to all electrical playbacks. Pooled latencies of all test fish are exemplarily presented for playbacks P_A_ (B) and F_3_ (C). Grey lines indicate the expected latency distribution based on playback EOD-distribution. Bin size: 1 ms.

All animals showed a preferred latency response (echo response) as well as latency avoidance response to the electrical playback signals, i.e. the fish responded to a certain proportion of playback EODs by emitting a time-locked EOD (Fig 9). The preferred latency, or 'echo response', ranged from 19 to 25 ms and occurred in response to all electrical playbacks. The same was true for latency avoidance responses, which directly preceded the echo-response at around 15 ms after the playback signal (Fig 9). No consistent differences in the ratio between observed and expected latencies were found based on the different playback IDI-patterns.

**Fig 10:**
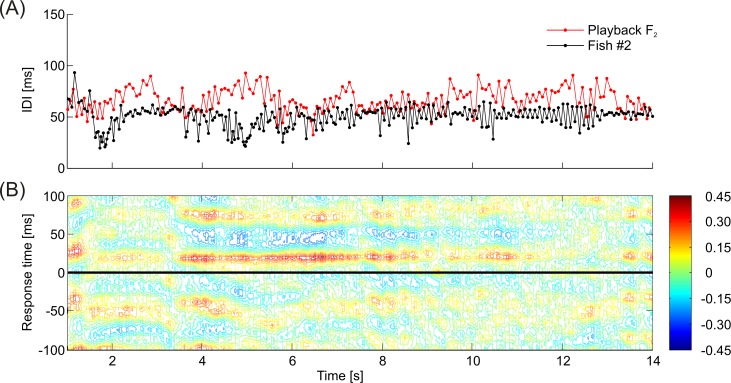
Adaptive cross-correlation analysis between pulse-sequences of playback and *M*. *rume*. (A) IDI-sequences of playback F_2_ (red) and an exemplary response of fish #2 (black). (B) Cross-correlation diagram for the sequence shown in (A). Correlation coefficients are plotted color-coded for response times of *M*. *rume* ± 100 ms in relation to the playback signals over the experimental time. The red band at a response time of about 20 ms in the upper part of the diagram in (B) demonstrates a relatively high correlation between the discharges of fish #2 and the dummy fish at this latency and indicates that the test fish synchronized its discharge activity to the playback for a time period of several seconds.

**Fig 11:**
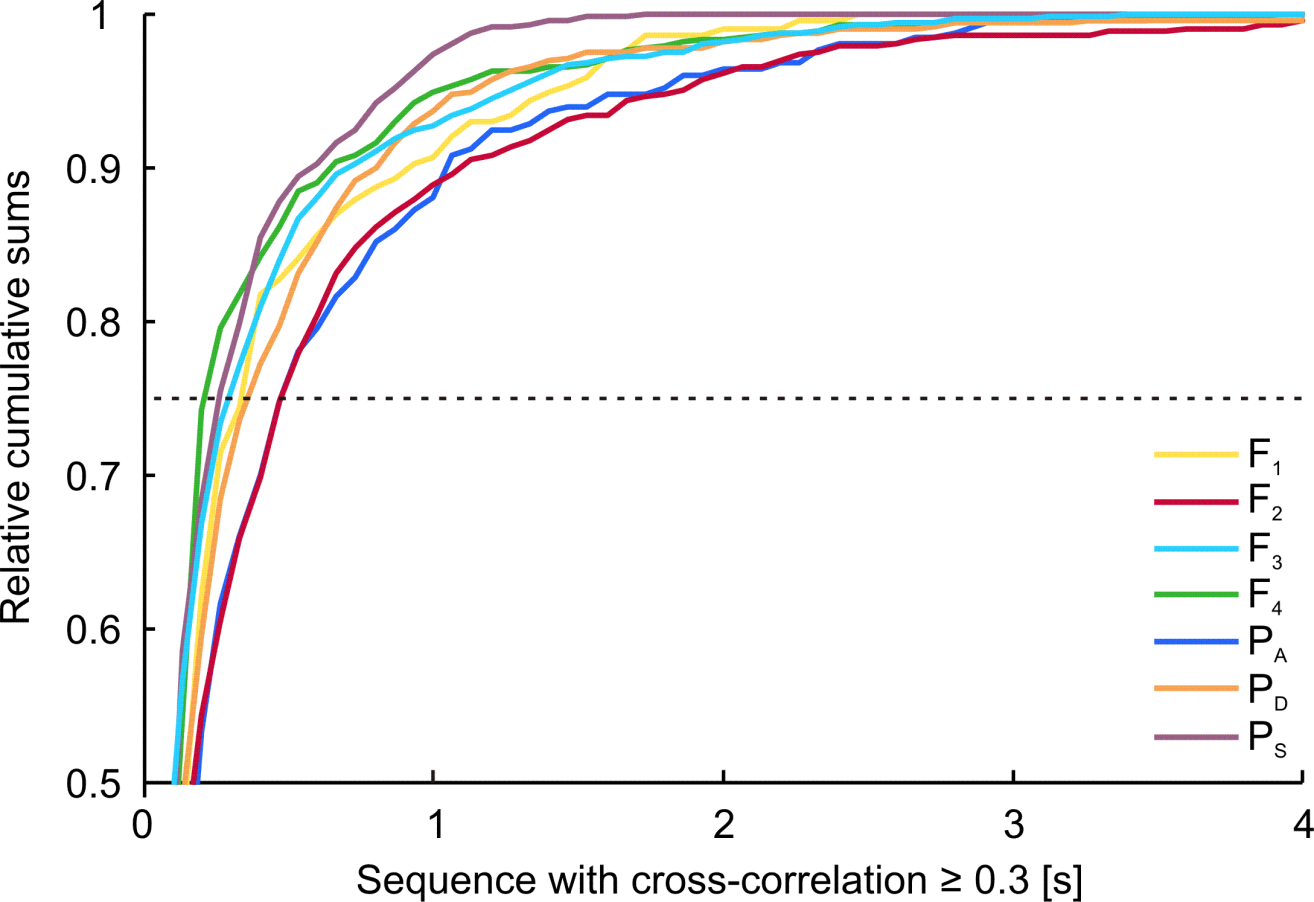
Sequences of electrical discharge interactions. Relative cumulative sums (RCS) of time periods with a cross-correlation coefficient ≥ 0.3. The graphs illustrate the proportions of sequences of a given length based on playback condition for temporal correlations between EODs of *M*. *rume* and the mobile dummy fish. Statistical comparisons between the effects of the different playbacks were performed at a RCS of 0.75. At this value, 75% of all sequences were shorter than the x-axis intersection of their respective graph with the dashed line.

Adaptive cross-correlations between the signal sequences of the playback and the EOD-sequence of the fish revealed that animals frequently synchronized their discharge activity to the playback signals, preferably at a response time of approximately 20 ms (Fig 10). The relative amount of maximum cross-correlation was on average significantly higher (*F*(1,97) = 171.030, *p* < 0.001) when IDI-sequences of test fish and dummy were recorded in the same trial (*mean* = 0.204, 95% CI [0.199, 0.210]) compared to randomly occurring correlations, which were calculated from fish IDI-sequences and playback patterns that were recorded during independent experimental trials (*mean* = 0.151, 95% CI [0.146, 0.157]). However, the difference in the relative amount of maximum correlation between the responses to the different playbacks after subtraction of randomly occurring correlations for each of the playback conditions accounts at the most for a statistical trend (*χ^2^*(6) = 11.571, *p* = 0.072). No matter which playback sequence was used, fish always synchronized a certain fraction of their EODs to the signals emitted by the dummy. This indicates that *M*. *rume* synchronized its discharge behavior largely independently of the current playback sequence and without adopting the actual patterns or frequency distributions of the particular playback. The duration of sequences with correlations between the signals of *M*. *rume* and the electrical playback exceeding 0.3, however, varied depending on the presented playback sequence (Fig 11). Longer runs of high correlation were elicited by playbacks F_2_(red) and P_A_ (dark-blue), whereas the low frequency playbacks F_4_ (green) and P_S_ (purple) accounted for fewer long sequences of high correlation. The influence of playback condition on the duration of periods of high correlation was statistically significant at a relative cumulative sum (RCS) of 0.75 (*χ^2^*(6) = 22.393, *p* = 0.001, dotted line in Fig 11).

### Motor interactions with the dummy fish

**Fig 12:**
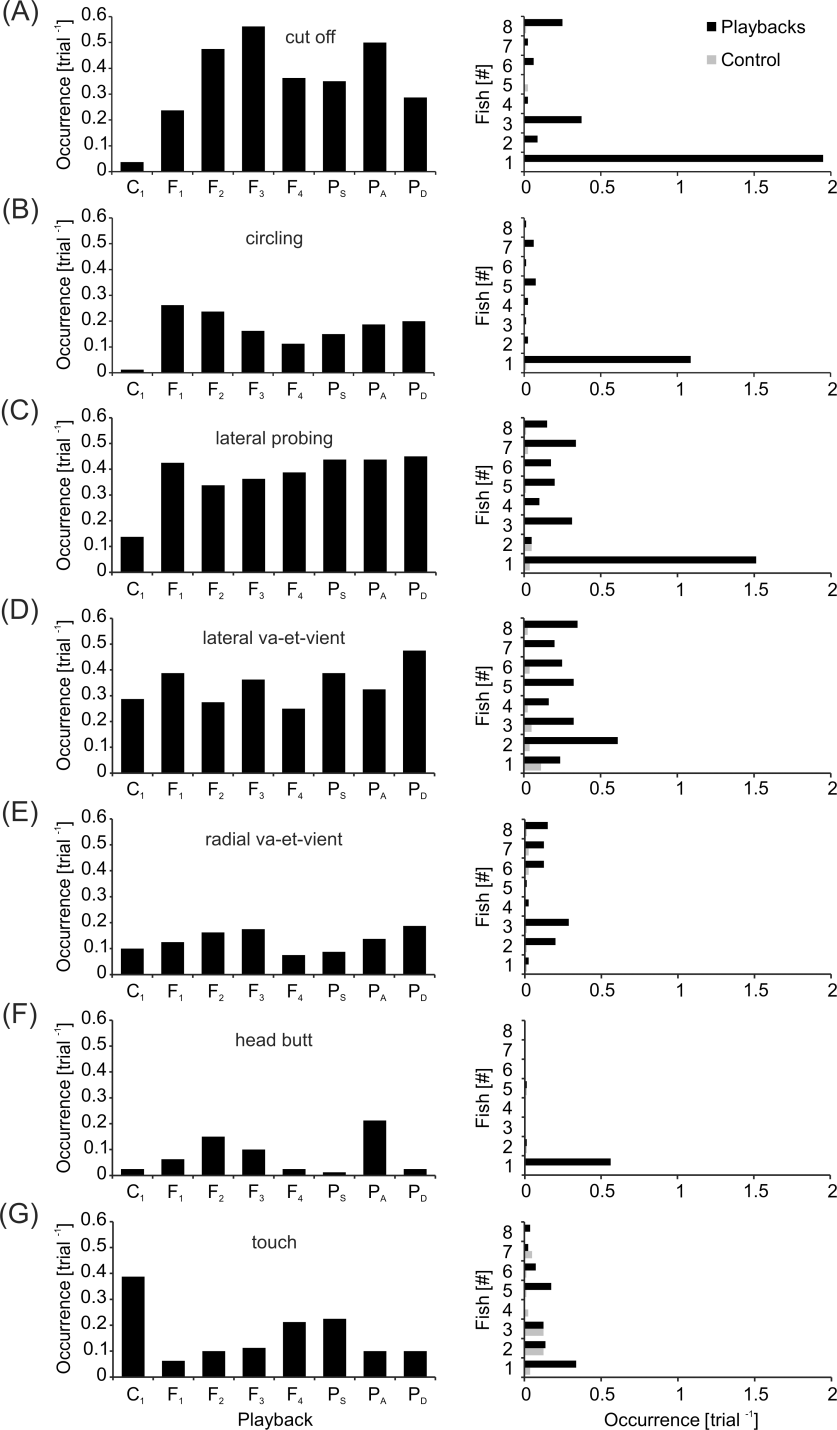
Motor behaviors of *M*. rume in response to the moving dummy fish. Seven types of behaviors were analyzed: (A) cut off, (B) circling, (C) lateral probing, (D) lateral va-et-vient, (E) radial va-et-vient, (F) head butt and (G) touch. The number of instances per trial depending on experimental condition is shown on the left. On the right hand side, the same number is resolved for all tested animals according to their relative rank within the hierarchy for all playback conditions (black) and the control C_1_ (grey).

In order to analyze the influence of electrical playbacks on interactive behaviors of *M*. *rume*, seven different motor patterns were quantified depending on experimental conditions (Fig 12, S1 Video). Statistically significant differences between the experimental conditions were detected for 'cut off' (Fig 12A, *χ^2^*(7) = 14.968, *p* = 0.036) and 'circling' (Fig 12B, *χ^2^*(7) = 15.817, *p* = 0.027). In both cases, almost no instances of the respective motor patterns occurred in response to the silent control condition C_1_, and the vast majority was performed by the most dominant fish #1. A similar overall response pattern was detected for 'lateral probing' (Fig 12C), although these differences were not statistically significant (*χ^2^*(7) = 7.314, *p* = 0.397). Both 'lateral-' (Fig 12D) and 'radial va-et-vient' (Fig 12E) were performed by all tested individuals, and occurred independently of experimental condition (*χ^2^*(7) = 11.189, *p* = 0.131; *χ^2^*(7) = 7.520, *p* = 0.377). 'Head butts' directed at the dummy fish (Fig 12F) came almost exclusively from the most dominant fish #1, and most instances were observed in response to playback P_A_, which featured discharge accelerations associated with aggressive behavior. Interestingly, most instances of touching the dummy fish were observed during the silent control C_1_, although the overall model for 'touch' (Fig 12G) was not significant (*χ^2^*(7) = 11.137, *p* = 0.133).

**Fig 13:**
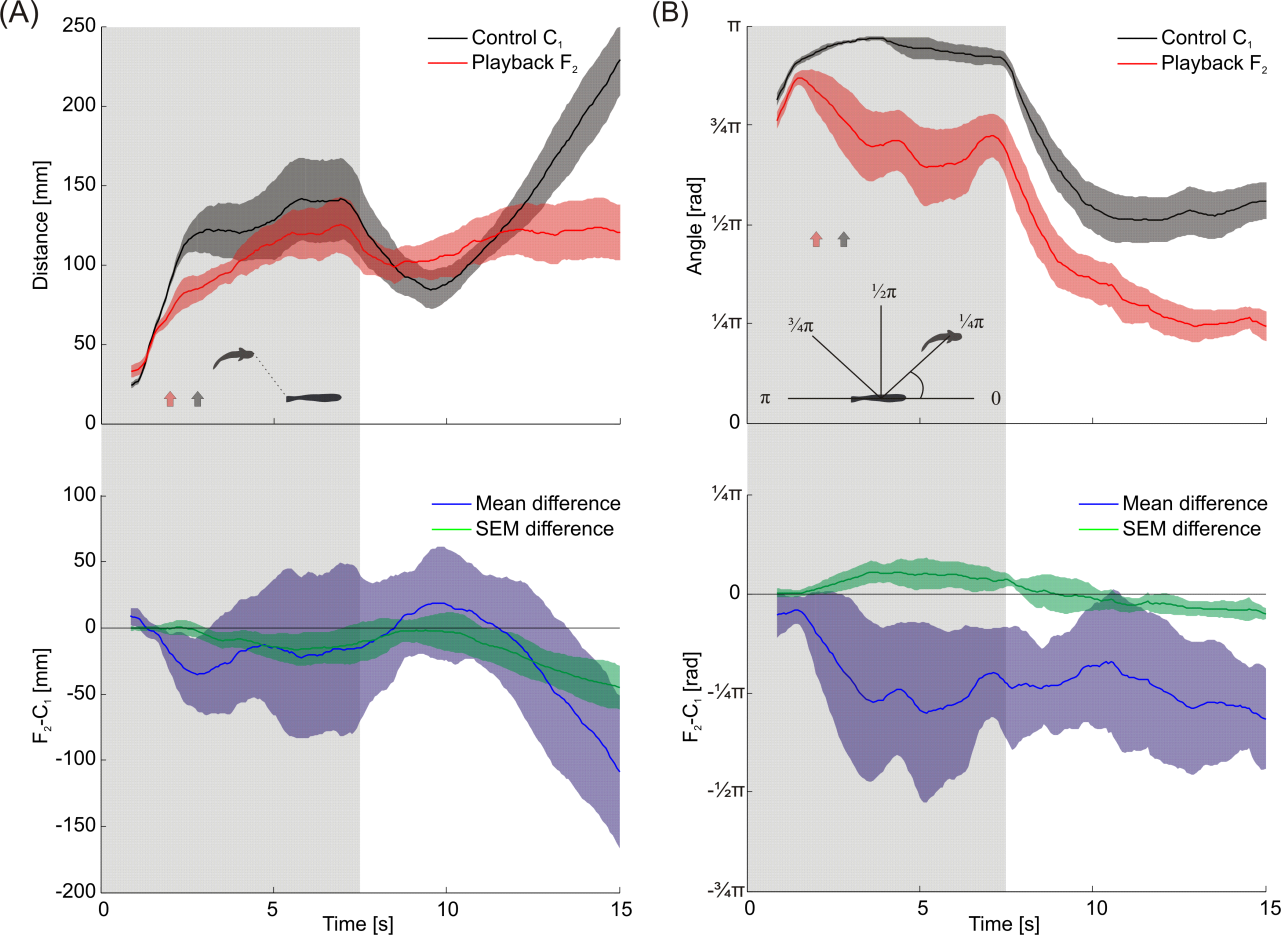
Spatial interactions of *M*. *rume* with the mobile dummy. (A) Distance between the focal fish's snout and the closest point on the dummy in the time course of all experiments with playback F_2_ (red) and the silent control C_1_ (black). Means and standard errors are depicted for all eight *M*. *rume* performing ten trials each. Differences between means (blue) and standard errors (green) between the two conditions are depicted in the section below, where 95% confidence intervals indicate that fish stayed longer in the vicinity of the dummy during playback presentation compared with the silent control C_1_.(B) Absolute angular difference between the direction from dummy to focal fish and the dummies orientation during the time course of all experiments with playback F_2_ (red) and control C_1_ (black). Mean values and the respective standard errors are depicted framewise. Differences between means (blue) and standard errors (green) between the conditions are presented with 95% confidence intervals in the section below, indicating that fish followed differently based on whether electrical playback signals were present or not. Arrows mark the average time when animals entered the testing area during playback presentation (red) and control (black). Shaded areas represent the time frame during which the dummy fish was moving.

Distance and angular relationship between dummy fish and the following *M*. *rume* was analyzed framewise over the time course of all experiments with the silently moving control C_1_ and for playback F_2_. The distance between the snout of the test fish and the closest point on the dummy fish is plotted for both conditions on the upper panel of Fig 13A. On average, fish followed faster (see Fig 3C) and closer during playback presentation compared to the control condition. Without playback presentation, the distance between fish and dummy was larger and consistently more variable, as indicated by the mean difference of standard errors in the lower panel of Fig 13A. After the dummy fish stopped moving, test fish approached closer, but moved away quicker during the control, whereas they stayed closer to the dummy fish when it emitted electrical playback signals.

The position of the following fish from the dummies coordinate system is visualized in Fig 13B by plotting the absolute angle between the dummy fish's direction of movement and the line connecting the centers of dummy and test fish over experimental time. While test fish tended to swim behind the dummy fish during the control condition, they followed on average more lateral and with a higher variability during playback presentation. The mean differences of means and standard errors depicted for both treatments in the lower panel suggest that these difference in following-behavior were consistent and depended on whether electrical playback signals were present or not.

**Fig 14:**
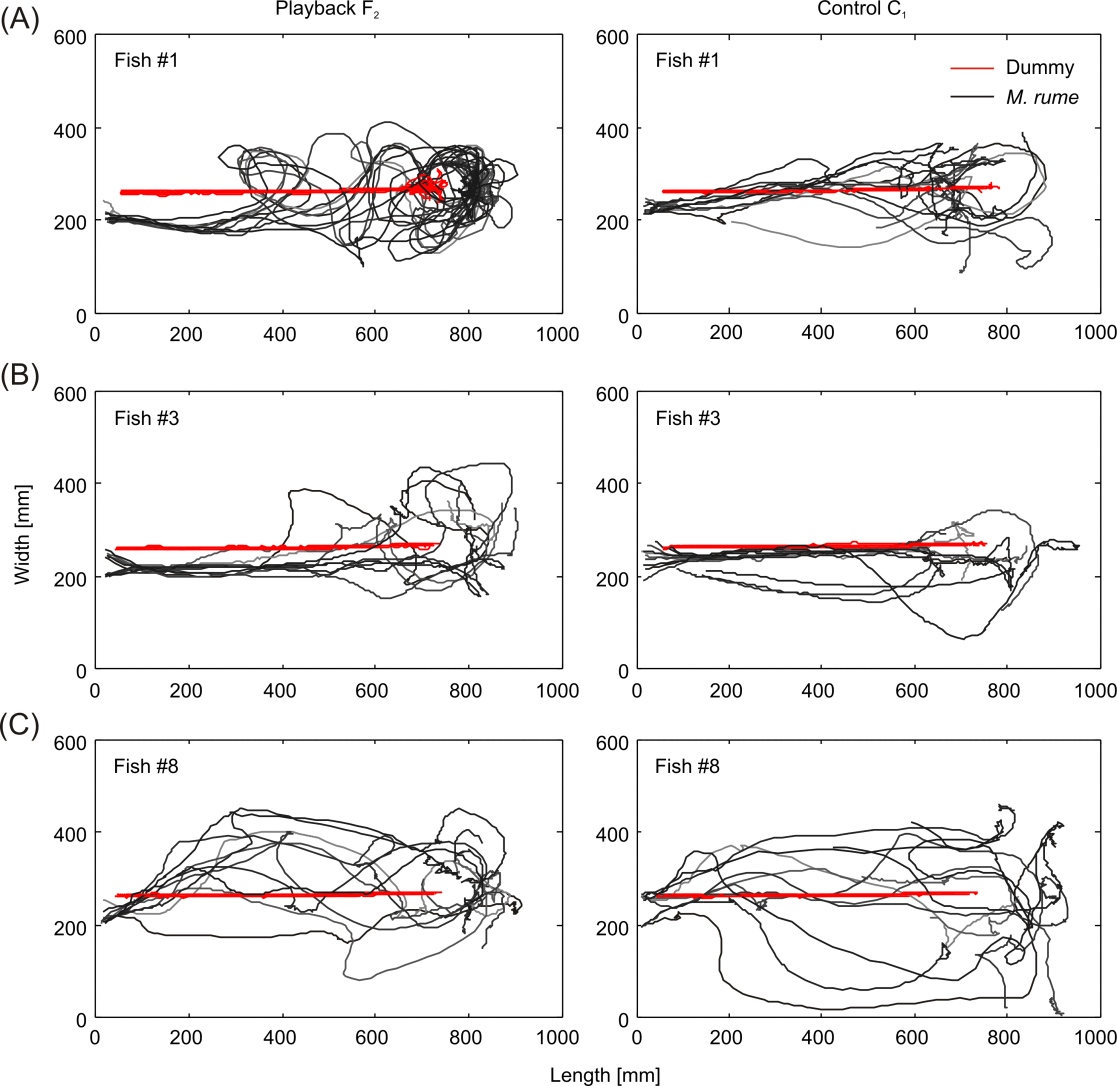
Swimming trajectories. Comparison of trajectories of dummy (red) and *M*. *rume* for 10 trials (grey, graded to distinguish trajectories from different trials) per condition in 3 fish (#1, #3 and #8) in response to playback F_2_ (left) and the electrically silent control C_1_ (right).

Similarities and differences in following-behavior between individual fish are further emphasized by the trajectories shown in Fig 14. During playback presentation, the most dominant fish #1 (Fig 14A) showed numerous instances of circling around the dummy fish both while it was moving and at its terminal position. Fish #1 always entered the testing area in parallel to the dummy fish's trajectory in the playback condition, but moved along the trajectory if the dummy moved without emitting electrical playback. This latter behavior was particular obvious in fish #3 (Fig 14B), which reproduced the dummy fish's trajectory quite closely during the control condition, but turned away soon after it stopped moving and swam back to the living area. The lowest ranking fish #8 generally kept a larger distance to the dummy fish, but approached closer during playback presentation than during the control condition (Fig 14C). Based on a Related-Samples Wilcoxon Signed-Rank Test, the average swimming speed of following *M*. *rume* in response to playback presentation (S4 Fig) was significantly higher during the first section of the experiment when the dummy fish was moving, compared to the response to the silent control (*z* = 2.100, *p* = 0.036). No significant difference in swimming speed between the conditions could be detected in the second half of the experiment, when the dummy fish had stopped moving (*z* = -0.980, *p* = 0.327).

**Fig 15:**
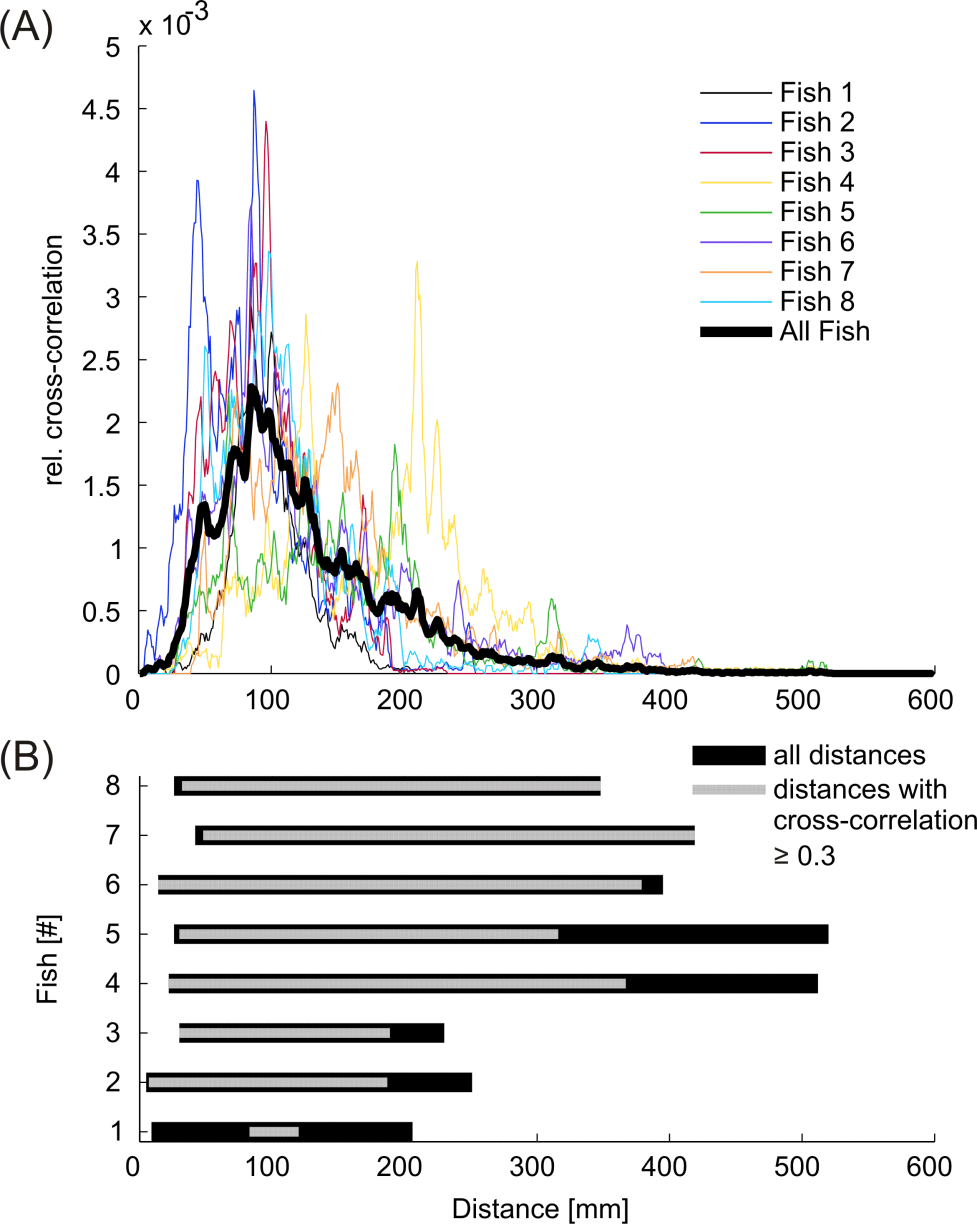
Influence of distance on interactive signaling. (A) Relative amount of correlation between discharge events of individual *M*. *rume* and playback F_2_ depending on the distance between the animal's snout and the closest point of the dummy fish. On average (thick black line) correlation was highest when fish and dummy were approximately 90 mm apart. (B) The total range of distances kept between each fish and the mobile dummy (black) is contrasted with the range of distances where correlations between the discharges of *M*. *rume* and playback F_2_ exceeded 0.3 (grey).

The simultaneous recording of electrical discharges and swimming behavior allowed to associate interactive signaling activity during discharge synchronizations with the spatial parameters obtained from swimming trajectories. The relative amount of correlation between the signals of *M*. *rume* and the mobile dummy fish was on average highest at a distance of approximately 90 mm during the presentation of playback F_2_ (Fig 15A). The longest distance of 520 mm was recorded between fish #5 and the mobile dummy fish. Correlation coefficients exceeding 0.3 occurred only up to a distance of 419 mm (fish #7, Fig 15B).

## Discussion

Our results provide new insights into the relationship between motor behaviors and electric signaling strategies in weakly electric fish. We show that a mobile fish dummy can recruit solitary individuals of *Mormyrus rume proboscirostris* from a shelter into an open area. This following-behavior was reliably observed in response to a variety of electrical playbacks with natural IDI-sequences and was, to a much smaller extent, also induced by the electrically silent control (Fig 3). It occurred despite the fact that visual perception of and orientation towards the dummy were not possible because of the experimental design. During fish-dummy interactions, animals frequently displayed stereotyped motor behaviors towards the dummy, and some of these patterns were almost only observed in fish that followed a dummy emitting electrical playback (Fig 12). This suggests that the playback-emitting, mobile dummy fish successfully induced an artificial social context, which can be used in the future by scientists to reveal behavioral principles in standardized and controlled experiments on electrocommunication. The presence of electrical playback also affected the spatial relationship between *M*. *rume* and the mobile fish dummy during following-behavior, thereby providing clues concerning the sensory systems involved in the observed behavior. The shift towards a lateral following-position in the presence of electrical playback (Fig 13B) indicates that the perception of EODs via the knollenorgan pathway serves not only in detecting communication signals of other fish, but also as an important sensory basis for spatial aspects of social interactions.

Our study also demonstrates the electric signaling strategies based on discharge frequencies, patterns and interactions that fish use when they follow a mobile dummy, which emits electrical playback. The electrical playback sequences used in this study were recorded from freely behaving individuals of *M*. *rume*, which were engaged in different behavioral contexts, such as aggressive interactions, hiding, foraging, slowly swimming and others (see above). As a consequence, the sequences used varied in average discharge frequencies (F_1_ –F_4_) and temporal IDI-patterns (P_S_, P_A_, P_D_). However, since these were exemplary recordings, one has to be careful to describe these sequences as typical for a certain behavioral context [68]. Different playbacks did not lead to different inclinations of the fish to follow the mobile dummy, with the exception of the low frequency playback F_4_. With this playback, lower ranking individuals were less likely to be recruited, a correlation that was also observed for the electrically silent control C_1_ (Table 1). A possible explanation for this correlation might be a potential relationship between dominance and personality traits of the tested individuals. Animals with a higher rank might have expressed a bolder personality profile and therefore reacted with a stronger tendency to explore the dummy fish during the more subtle stimulation during the low-frequency playback F_4_ and the electrically silent control condition C_1_ [69].

Overall discharge frequencies and IDI-distributions of the following fish were mostly unaffected by the presented playback sequences. Similarly, interactive signaling, such as producing echo responses to the playback EODs, was observed as a response to all playbacks. However, context dependent communication was obvious at the level of temporal pattern generation. By associating the electrical responses of following fish with the relative dominance rank between individuals, particularly double pulses could be identified as a signaling pattern that was displayed with communicative intent. In order to interpret these results, one has to take into account that mormyrids simultaneously employ their electric signals for active electrolocation and electrocommunication.

A possible strategy for mormyrids to communicate behavioral states during electrocommunication could be to adopt a similar overall discharge behavior as a conspecific, which should become manifest in a shift of an animals' IDI-distribution towards the one emitted by the dummy in our experiments. A multitude of studies on several mormyrid species have established that variations in overall IDI-distribution depend on activity level and behavioral context of weakly electric fish [23, 26, 28, 70]. In a study with the mormyrid *Gnathonemus petersii* using stationary playback electrodes emitting sequences recorded in different behavioral contexts (aggression or resting), the receiving fish responded with IDI-sequences of varying overall discharge frequencies [41]. This was not the case in the current study. Different playbacks did not lead to predictable differences in overall IDI-distribution of the following fish. Although individual differences in IDI-distribution were observed between different *M*. *rume*, the resemblance of the overall distribution patterns of the following fish was always closest to playback F_2_, which was originally recorded from a *M*. *rume* following an electrically silent dummy fish (Fig 4). Only in the stationary control condition C_2_ (S1 Fig), there was a tendency of the fish to use longer IDIs and a broader interval distribution, suggesting a general effect of the moving dummy on discharge frequency and regularization that persisted independently of electric playback presentation. It therefore appears unlikely that in our experiments intentional communication of a particular behavioral context occurred at the level of overall discharge frequency. In our experiments, the dummy displayed a stereotypical, constant behavior of swimming within 7.5 s from the starting to the end position in a straight line and always at the same speed, regardless of the playback condition. After stopping, it continued emitting the particular playback sequence for another 7.5 s. As a consequence a discrepancy might have occurred between the dummy’s behavior and its EOD signaling: even if the playback sequences contained information about the original behavioral context during the recordings, the behavior of the dummy was always just straight line swimming. If the dummy were a real fish and its locomotor behavior corresponded to its signaling, the test fish might have also adjusted their overall discharge frequencies. Instead, they followed the dummy and emitted a typical 'following pattern' which resembled the pattern F_2_. They thus would have communicated their current behavioral state, which was ‘following’. Our results suggest that IDI-distributions of the following fish were mainly determined by other needs, such as active electrolocation when following the dummy. Nevertheless, changes in overall discharge frequency may still provide eavesdropping individuals with information concerning a conspecifics current activity, which was invariable in our experiments.

A second possible strategy in electrocommunication involves interactive signaling patterns, for example in the form of echo-responses or discharge synchronizations, which could in turn also result in a similar IDI-distribution of the playback and the tested fish. Such interactive signaling responses by the recruited fish were elicited by all playback types. The analysis of cross-correlations between playback pulses and the timing of EOD responses in *M*. *rume* showed that animals interacted electrically with the dummy largely independently of similarities between the IDI-distributions of fish and playback (Fig 10). While no differences between treatments remained after subtraction of randomly occurring correlations, and overall correlation coefficients were not very high in general, some playback patterns elicited on average longer periods of relatively high correlation compared to others (Fig 11). It is therefore possible to visualize the time course of EOD-synchronization and thereby conclude on the behavioral situations where they occurred. Most correlations were prominently found at a response time of approximately 20 ms after a playback EOD, which corresponds to previous descriptions of the latency of the echo response in *M*. *rume* [37]. In this study, preferred latency responses were observed in all tested individuals and in response to all presented playback patterns, although the degree of pronunciation was variable. In addition, all animals showed preferred latency avoidance within an interval directly preceding the echo response (Fig 9). This stands in contrast to results by Lücker and Kramer (34) who found that male and female *Pollimyrus isidori* reacted differently by displaying either a preferred latency response or preferred latency avoidance. Exhibition of preferred latency responses and preferred latency avoidance has been reported to occur in both male and female *Mormyrus kannume*, although not within the same individuals [71].

The third and most obvious electrocommunication strategy in mormyrid weakly electric fish is to encode communicative intent into certain patterns within discharge sequences. Such patterns were represented in this study by discharge regularizations and double-pulse patterns. Regularizations of electric discharge activity have been suggested to increase the spatiotemporal resolution of active sensing and lead to constant sensory input at the receptor level, thus improving the performance of active electrolocation [24, 72]. Regularizations have, however, also been described in a communicative context as a response to electrical stimuli [21] and as a reaction to stimulation with conspecific signals [22, 25]. While [21] hypothesized that regularizations improve active electrolocation, reports of regularized intervals during antagonistic behavior [73] and during courtship and spawning [29, 74] suggest, that this pattern may also have communicative value. In this study, IDI-regularizations were quantified using autocorrelation of intervals within a 200 ms time frame (Fig 8). With the exception of playback F_1_, *M*. *rume* displayed stronger regularization in response to higher discharge frequencies contained in the playback sequence, which is similar to the findings by [21]. Quantification over time allowed distinguishing between the effects of the dummies movement and the presentation of various electrical playbacks on the propensity of *M*. *rume* to regularize IDIs. Since the strength of regularization peaks after the offset of playback presentation, it seems unlikely that the observed behavior is solely performed to improve active sensing. It appears therefore plausible to presume communicative intent associated with strong regularization patterns in a social context.

The communicative nature of double pulses is less ambiguous than that of simple discharge regularizations. Double-pulse patterns have been described as alternating long and short IDIs in several mormyrid species and can be classified as a form of regularization themselves [22]. They have mainly been observed within antagonistic contexts and during aggressive behavior in *G*. *petersii* [28, 73, 75], and are considered to be aggressive threat signals, which are also displayed by nest-guarding males in two *Pollimyrus* species [29]. In *M*. *rume*, it has previously been observed that double-pulse patterns were emitted by solitary individuals only in response to electrical playback presentation [76]. The present study confirms this result by demonstrating that double pulses were virtually absent in response to the control condition (Fig 6). Additionally, most double pulses were emitted in response to the playback pattern P_D_, which also contained double pulses. Since the emission of double pulses was subject to habituation, and there appears to be no obvious advantage for active electrolocation, we suggest this pattern to serve as a threat signal in *M*. *rume* as well, although rather with respect to claiming dominance at the beginning of a sequential assessment strategy [77] than in relation to overt aggression. This assumption is supported by the observation that higher ranking individuals produced higher amounts of double pulses (Fig 7). Although our observation periods were much too short to observe the processes underlying hierarchy formation, these results open the opportunity to test the communicative value of this signal pattern by systematic variation of its properties in controlled playback experiments or in dyadic contests.

Communication displays ultimately aim at triggering behavioral responses of the receiving party and may thereby initiate some form of physical interaction between signaler and receiver. The motor patterns displayed by *M*. *rume* towards the mobile dummy fish lend further support to the idea that interactive behaviors between live fish and a playback emitting dummy can be used as a proxy for the investigation of social behavior in mormyrids under controlled experimental conditions (see also [78]). Trajectory 'cut offs', complete 'circling' and 'head butts' were hardly directed at the electrically silent dummy fish. This shows that appropriate locomotor communication behaviors are only provoked by dummies emitting EODs (Fig 12). Both 'head butts' and 'circling' have previously been described in social interactions between mormyrids [26]. These results thus provide a framework for further studies involving interactive playback patterns, as well as more complex trajectories [56].

The inter-individual distance at which a signal occurs between weakly electric fish may also allow concluding whether its function relates to active electrolocation or electrocommunication. The active range of electrocommunication has been inferred from experimental results [25, 79] and extends beyond the limits of active electrolocation due to the high sensitivity of the knollenorgan receptors [80]. Double pulses and high correlations with the playback signals emitted by the mobile dummy fish occurred up to a distance of 287 and 419 mm (Fig 15), respectively, which is approximately within the range where discharge cessations were observed in response to an approaching conspecific in *Brienomyrus niger* [79]. The highest amount of both signaling types, however, was most prominent at a distance of 90–100 mm, which corresponds to the outer limit of active electrolocation [81].

The capability to locate the source of a signal is crucial if the objective of communication is to initiate social interactions. Similar studies aiming at manipulating the behavior of other, non-electric fish species by using mobile dummy fish have mainly relied on visual cues, or at least made no explicit assumption concerning the sensory systems involved in triggering the observed behavior [52, 54, 82, 83]. Since all our experiments were performed in darkness with only infrared illumination, vision can be excluded to have mediated following-behavior [62, 84]. Although not much is known about its efficiency, mormyrids also possess a functional lateral line system [85], and the fact that animals tended to follow right behind the mobile dummy fish, often reproducing its trajectory, during the silent control C_1_ (Fig 14), might suggest an involvement of hydrodynamic cues in following-behavior [86]. Lateral line information has been demonstrated to play a part in shoaling behavior [87, 88], and hydrodynamic cues produced by robotic fish have been shown to influence swimming preferences in individual fish [46, 89]. In the present study, animals also had their active electric sense at their disposal, which could have been employed to detect the dummy fish within the range of active electrolocation [90]. The fact that fish were following the EOD-emitting dummy mainly in a lateral position (Fig 13B) suggests that electric signals may be in general a natural determinant of spacing between individual weakly electric fish [91], and that passive electroreception, i.e., the perception of the EODs of a conspecific, may be more relevant for following than hydrodynamic sensing and active electrolocation. This assumption is supported by the findings of Schluger and Hopkins [92], who demonstrated, that weakly electric fish navigate along the electrical field lines in order to approach an electrical dipole source such as a conspecific individual emitting EODs.

Given the many overlaps in both electric signaling behaviors and motor response patterns that are directed either at inanimate objects during active electrolocation or towards conspecific individuals during social encounters, it may on many occasions be neither possible nor reasonable to attempt assigning a particular behavior exclusively to either active electrolocation or electrocommunication. Lateral probing during active electrolocation and circling during social interactions may not be fundamentally different behaviors [19], and it is easy to conceive, how regularization patterns, which may have evolved to improve active sensing, take over some communicative function by means of ritualization [64]. A similar transition from a pure electrolocation feature to a system involving a communicative function could have occurred for interactive signaling patterns. Echoing, which can be a means to avoid the jamming of an animal's sensory perception during active electrolocation [36], also leads to synchronized bursts between individuals and thus may serve in mutual recognition and group coherence [35]. Synchronization of EOD timing with a conspecific may therefore be a means to address another individual without impairing the functionality of active electrolocation in the process.

Communication systems can develop over evolutionary times when sensory cues, inadvertently generated by animals without communicative intent, allow conspecific individuals to predict the behavior of the animal generating the cue by exploiting pre-existing sensory systems [1]. Although encoding 'conventional signals' [93] into IDI-sequences appears plausible from a theoretical point of view, the actual amount of distinct signal patterns that can be produced may be limited due to the properties of the nuclei involved in central pattern generation in the mormyrid brain [94]. The difficulty to isolate unequivocal communication features from overall IDI-distributions, as well as the sometimes gradual transition between electrolocation and electrocommunication signals emphasize the dual nature of electric signaling in weakly electric fish. Similarly in bats, dual functions of vocalization for both echolocation and social communication have recently been reported [95–97]. Between simple eavesdropping, during which individuals could deduce a conspecifics behavior by monitoring its discharge rate, and encoding conventional information into stereotyped IDI-patterns with communicative intent, electrocommunication may rely on more subtle interactions whose true significance has yet to be uncovered.

## Supporting information

S1 Data Matlab structure array containing all analyzed EOD time series of *M*. *rume* and the respective playback sequences. (RAR)

S1 Fig Box plots indicating means, medians, modes and inter-quartile differences of the IDI-distributions of eight *M*. *rume* in response to different electrical playback conditions and controls. (TIF)

S2 Fig Average of maximum autocorrelation within a time frame of 200 ms over a 15 s recording period for each experimental condition (*mean ± s*.*e*.*m*.). Categories sharing a common superscript differ based on Bonferroni adjusted *p* - values.
(TIF)

S3 Fig Relative cumulative sums of temporal sequences with a correlation coefficient ≥ 0.3. Graphs indicate the proportion of sequences with high autocorrelation for a given duration based on playback condition.
(TIF)

S4 Fig Swimming speed. (A) Average swimming speed (*mean* ± *s*.*e*.*m*.). The shaded area represents the section where the dummy fish was moving. (B) Bar chart comparing average swimming speeds in response to control C_1_ and playback F_2_ for different time sections of the experiment.
(TIF)

S1 Table Properties of the seven IDI-sequences that were used during playback experiments. (DOCX)

S1 Video Video examples of experimental trials featuring typical instances of the quantified motor interactions ‘cut off’, ‘circling’, ‘lateral probing’, ‘lateral va-et-vient’, ‘radial va-et-vient’, ‘head butt’ and ‘touch’. (MP4)
