# Supplementary figures and images for: Social interactions between live and artificial weakly electric fish: Electrocommunication and locomotor behavior of Mormyrus rume proboscirostris towards a mobile dummy fish
Source: PLoS One. 2017 Sep 13;12(9):e0184622. doi: 10.1371/journal.pone.0184622 (PMC5597219; doi:10.1371/journal.pone.0184622)

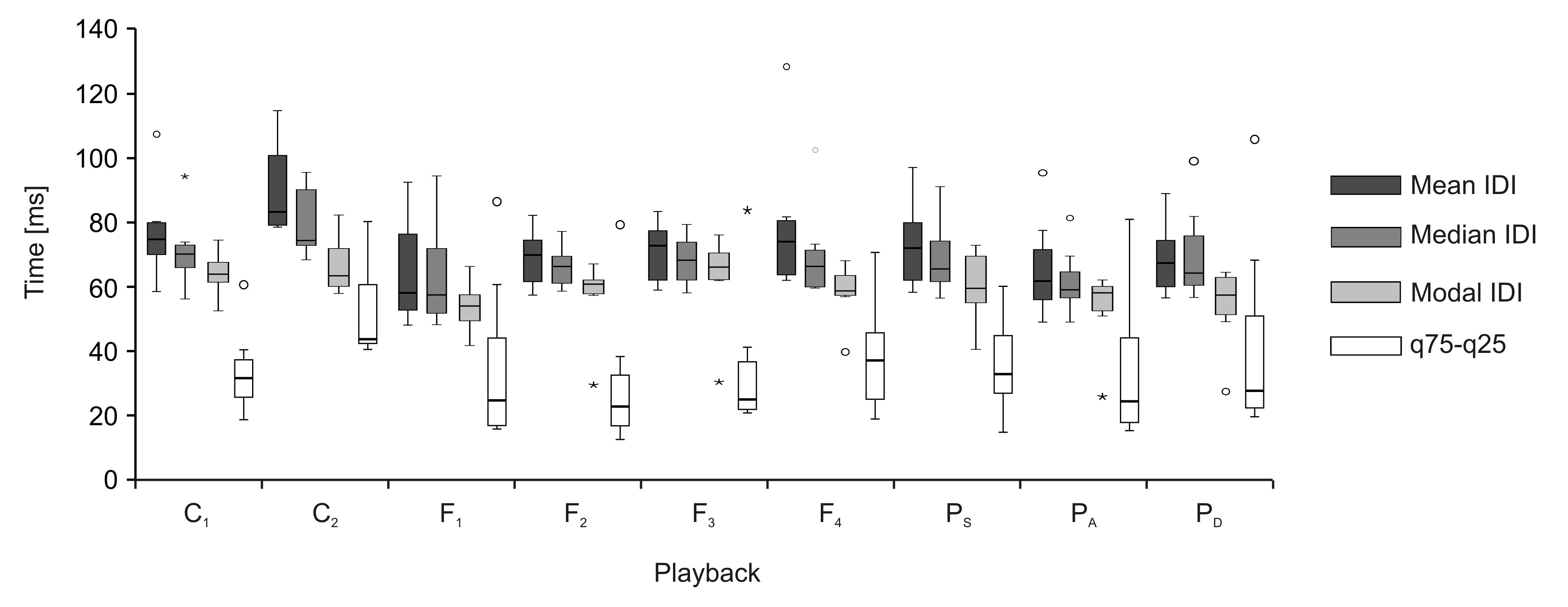

Supplement: S1 Fig — (TIF) [file pone.0184622.s002.tif]

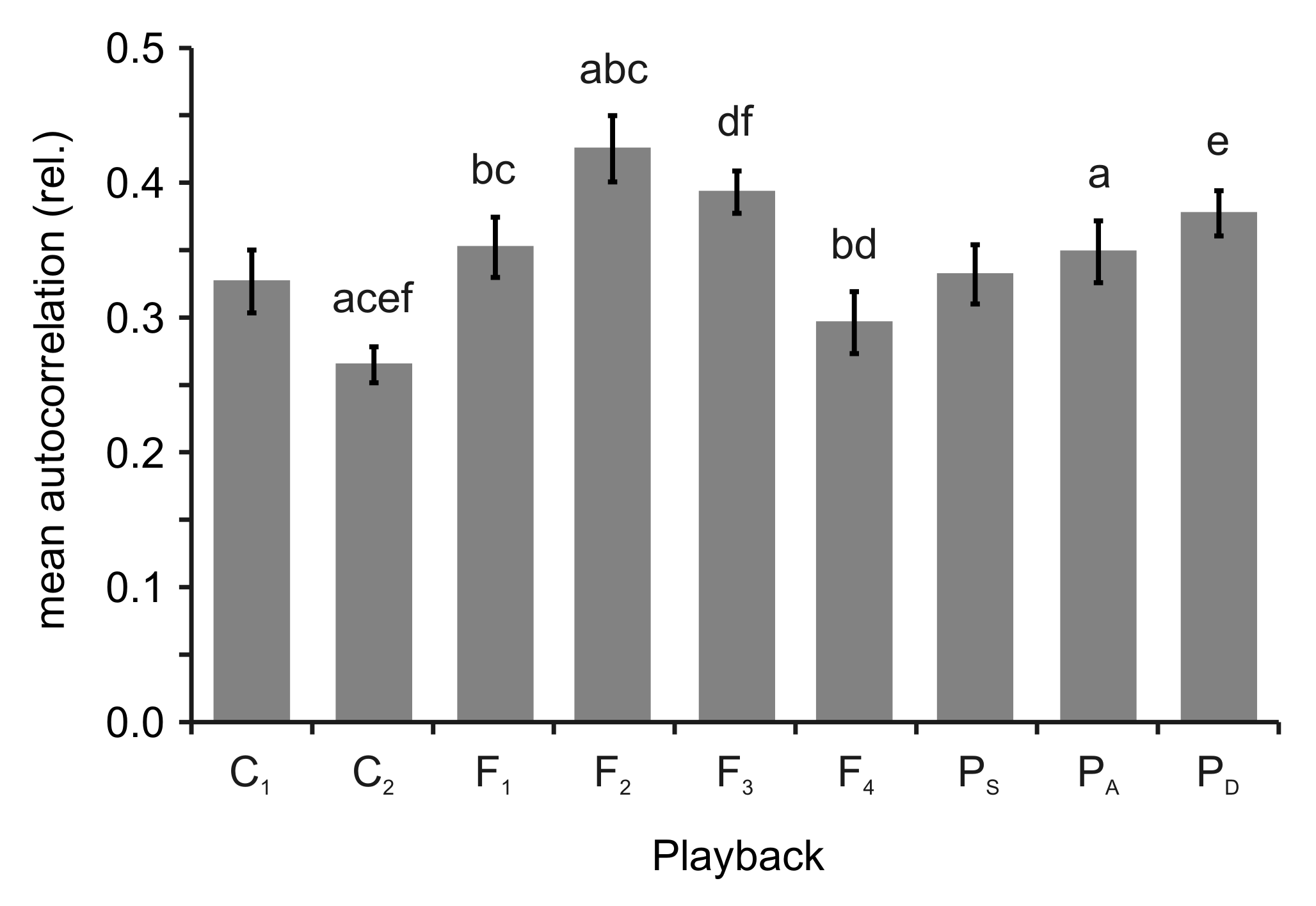

Supplement: S2 Fig — Categories sharing a common superscript differ based on Bonferroni adjusted p - values. (TIF) [file pone.0184622.s003.tif]
