# Supplementary material for: Social interactions between live and artificial weakly electric fish: Electrocommunication and locomotor behavior of Mormyrus rume proboscirostris towards a mobile dummy fish
Source: PLoS One. 2017 Sep 13;12(9):e0184622. doi: 10.1371/journal.pone.0184622 (PMC5597219; doi:10.1371/journal.pone.0184622)
Supplement: S1 Table — (DOCX) [file pone.0184622.s006.docx]

**S1 Table: Properties of the seven IDI-sequences that were used during playback experiments.**

| Playback | Context | min IDI | max IDI | IDI mode | IDI mean | Variance [ms] | Mean frequency | Variance [Hz] | Special |
| --- | --- | --- | --- | --- | --- | --- | --- | --- | --- |
| F_1_ | Foraging in a group | 17 ms | 80 ms | 31 ms | 34 ms | 91 | 31.0 Hz | 53.6 | --- |
| F_2_ | Following after a moving fishing bite | 33 ms | 93 ms | 64 ms | 65 ms | 127 | 15.8 Hz | 8.6 | --- |
| F_3_ | Slowly swimming | 44 ms | 150 ms | 94 ms | 94 ms | 329 | 11.1 Hz | 5.4 | --- |
| F_4_ | Resting | 67 ms | 308 ms | 260 ms | 239 ms | 1924 | 4.5 Hz | 2.9 | --- |
| P_S_ | Subordinate | 63 ms | 1356 ms | 149 ms |  |  | --- | --- | Cessations |
| P_A_ | Aggressive interaction | 17 ms | 101 ms | 26/50 ms |  |  | --- | --- | Accelerations |
| P_D_ | Reaction to playback signals | 20 ms | 109 ms | 22/44/91 ms |  |  | --- | --- | Double-pulses |
